# Aurora B prevents premature removal of spindle assembly checkpoint proteins from the kinetochore: A key role for Aurora B in mitosis

**DOI:** 10.18632/oncotarget.10657

**Published:** 2016-07-18

**Authors:** Mark D. Gurden, Simon J. Anderhub, Amir Faisal, Spiros Linardopoulos

**Affiliations:** ^1^ Breast Cancer Now, Division of Breast Cancer Research, The Institute of Cancer Research, London, United Kingdom; ^2^ Cancer Research UK Cancer Therapeutics Unit, Division of Cancer Therapeutics, The Institute of Cancer Research, London, United Kingdom; ^3^ Present address: Lahore University of Management Sciences, D.H.A. Lahore Cantt, Lahore, Pakistan; ^4^ Present address: Phenex Pharmaceuticals, Ludwigshafen am Rhein, Germany

**Keywords:** Aurora B, MPS1, mitosis, inhibition, SAC

## Abstract

Accurate chromosome segregation is dependent on the spindle assembly checkpoint (SAC). In current models, the key direct role of Aurora B in the SAC has been suggested to be to promote rapid kinetochore localisation of MPS1, allowing MPS1 to generate the checkpoint signal. However, Aurora B is also thought to play an indirect role in the SAC through the destabilisation of kinetochore-microtubule (KT-MT) attachments. Here, we demonstrate that Aurora B activity is not required for the kinetochore recruitment of the majority of SAC proteins. More importantly, we show that the primary role of Aurora B in the SAC is to prevent the premature removal of SAC proteins from the kinetochore, which is strictly dependent on KT-MT interactions. Moreover, in the presence of KT-MT interactions, Aurora B inhibition silences a persistent SAC induced by tethering MPS1 to the kinetochore. This explains the highly synergistic interaction between Aurora B and MPS1 inhibitors to override the SAC, which is lost when cells are pre-arrested in nocodazole. Furthermore, we show that Aurora B and MPS1 inhibitors synergistically kill a panel of breast and colon cancer cell lines, including cells that are otherwise insensitive to Aurora B inhibitors alone. These data demonstrate that the major role of Aurora B in SAC is to prevent the removal of SAC proteins from tensionless kinetochores, thus inhibiting premature SAC silencing, and highlights a therapeutic strategy through combination of Aurora B and MPS1 inhibitors.

## INTRODUCTION

The correct functioning of the spindle assembly checkpoint (SAC) is essential for maintaining genomic stability. Through monitoring kinetochore-microtubule (KT-MT) attachments, the SAC ensures cells do not separate sister chromatids prematurely. Central to SAC signalling is the recruitment of a network of proteins to unattached kinetochores, resulting in the formation of the mitotic checkpoint complex (MCC; CDC20/BUBR1/BUB3/MAD2), which inhibits the Anaphase Promoting Complex/Cyclosome (APC/C) and progression into anaphase [1].

Phosphorylation is a major mechanism for regulating protein activity and localisation during mitosis, two seminal kinases being Aurora B and MPS1. Aurora B forms part of the Chromosome Passenger Complex (CPC), which localises to the centromere during mitosis where it corrects erroneous chromosome-microtubule attachment errors, as well as regulating the SAC [2]. MPS1 localises to the outer kinetochore in early mitosis and is required for the recruitment of SAC proteins to the kinetochore throughout mitosis, as well as catalysing the formation of the MCC [3–6]. MPS1 also plays a role in chromosome alignment by competing with microtubules for binding NDC80 complex [1, 7, 8].

Substantial progress has been made in understanding the mechanism of the SAC and in the cross regulation between Aurora B and MPS1. It has been proposed that MPS1 regulates Aurora B through enhancing centromere Aurora B localisation at the onset of mitosis [9] and secondly, although controversial, MPS1 phosphorylates Borealin (part of the CPC), stimulating Aurora B error correction activity [10]. Likewise, the binding of MPS1 to HEC1 at the kinetochore is enhanced by Aurora B phosphorylation of both HEC1 and the MPS1 N-terminus. However, since MPS1 also binds to NUF2 independent from Aurora B, MPS1 kinetochore localisation and function is not strictly dependent on Aurora B activity [1, 8].

Despite the major advances in our understanding of the SAC, it still remains unknown whether Aurora B plays a role in the SAC distinct from enhancing MPS1 localisation and activity [11]. For instance, Aurora B activity is reported to be required for kinetochore assembly via the phosphorylation of ZWINT1, stimulating recruitment of the RZZ complex [12, 13]. However, MPS1 is reported to perform the same function [6]. This could suggest a partial redundancy between the two kinases, similar to recent reports of PLK1 and MPS1 [14, 15]. In yeast, Aurora B (Ipl1) is believed to primarily contribute to the SAC through creating unattached kinetochores [16], thus it has been argued that inhibiting Aurora B activity causes stable KT-MT interactions, which by itself is sufficient to allow SAC silencing, although this still requires further evidence [17]. However, recent papers in mammalian cells have argued that the key role of Aurora B in the SAC is to recruit of MPS1 to the kinetochore and thus all subsequent SAC proteins, thereby enhancing checkpoint production [11]. Work originating over a decade ago demonstrated that whilst Aurora B depletion/inhibition can override a taxol-induced SAC, nocodazole-arrested cells remained largely unaffected [18–20]. This difference does not seem to be adequately explained by Aurora B enhancing MPS1 localisation and checkpoint activation [11]. However, when cells are treated prior to mitotic entry, Aurora B inhibition synergises with low doses of MPS1 inhibitors to override a nocodazole-induced SAC [11, 21]. Nonetheless, these studies neither addressed, nor sufficiently demonstrated why Aurora B inhibitors alone can override the SAC when pre-arrested in taxol, but not in nocodazole. Furthermore, whilst the constitutive targeting of MAD1 to kinetochore is sufficient to cause a persistent metaphase arrest, this arrest could still be overcome using an Aurora B inhibitor [22], suggesting it may have additional functions in the SAC other than its initial prophase recruitment of MPS1.

In this study we aimed to directly compare and contrast the effects of Aurora B and MPS1 inhibition on the establishment and maintenance of the SAC, in order to establish they key role Aurora B plays in regulating the SAC. We provide evidence to show that Aurora B activity is essential to inhibit the premature removal of MPS1 and SAC proteins from the kinetochore (SAC silencing), which is dependent on KT-MT interactions. We then further examine the synergy between the two kinases and explore their potential use in anti-cancer therapy.

## RESULTS

### The effect of MPS1 and Aurora B inhibitors on the spindle assembly checkpoint

**Figure 1:**
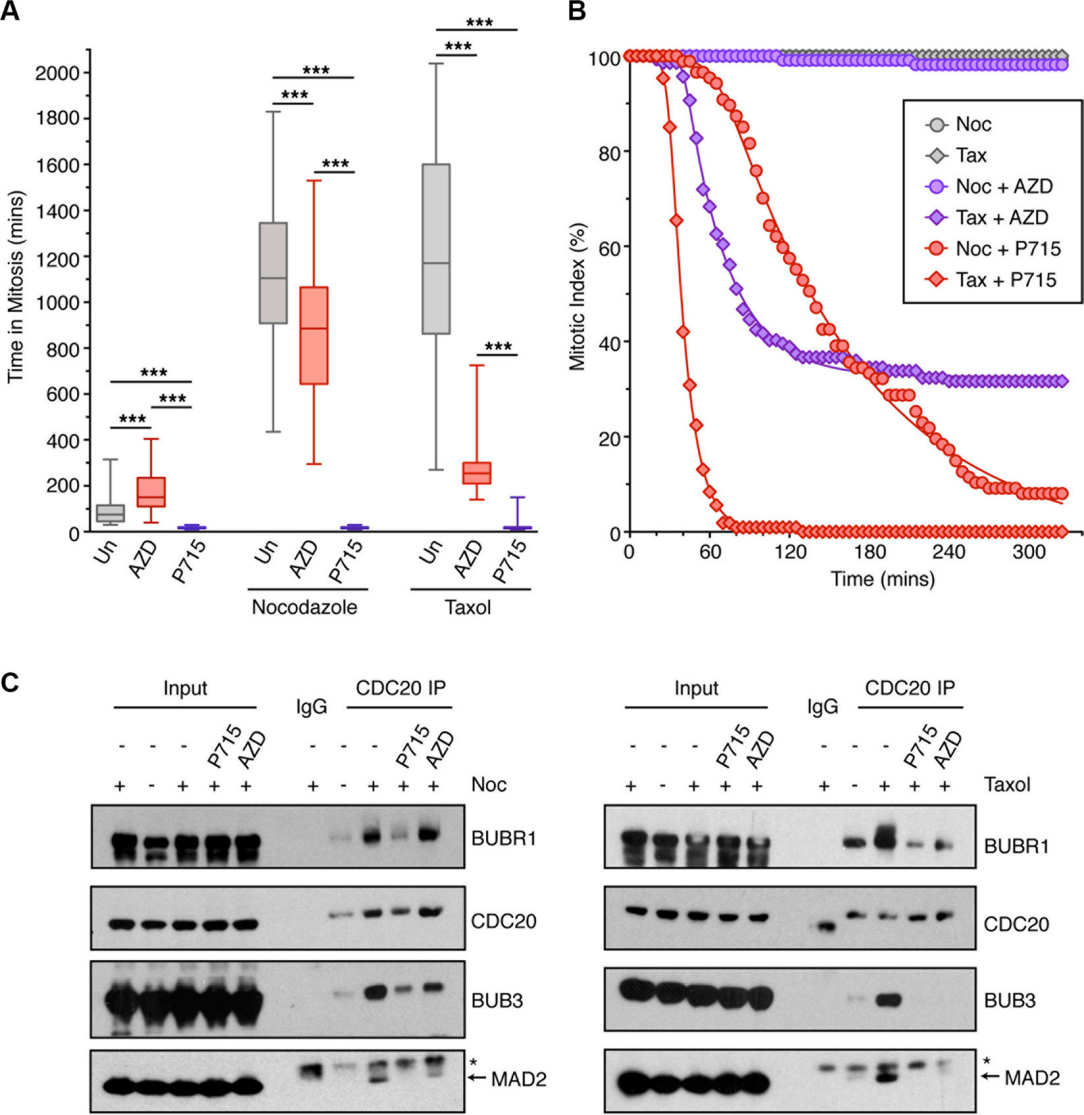
The effects of MPS1 and Aurora B inhibition on the SAC (**A**) Box-and-whisper plot showing the time HeLa cells (stably expressing Histone H2B-mCherry) spent in mitosis following treatment with the indicated drug (Un; untreated, AZD; 0.5 μM AZD1152, P715; 1.5 μM NMS-P715). The boxes represent the interquartile ranges and the whisker the full range. The result was analysed by One-way ANOVA with ^***^ indicating *p* < 0.0001. *N* = > 40 cells per condition. (**B**) Line graphs showing the mitotic exit of cells, analysed by time-lapse, pre-arrested for 18 hours in nocodazole (noc) and taxol (tax), then treated with 0.5 μM AZD1152 (AZD) or 1.5 μM NMS-P715 (P715) at 0 mins. *N* = > 87 cells per condition. (**C**) IP of CDC20 from HeLa cells arrested with nocodazole (noc; left panel) and taxol (right panel), then treated for 2 hours with AZD1152 or NMS-P715 and MG132. Lysates were analysed by immunoblotting. Asterix shows non-specific band for the MAD2 antibody.

To examine whether MPS1 and Aurora B kinases play similar, complementary, or unique roles in regulating the SAC, we examined the effect of kinase inhibitors on the mitotic timing of HeLa cells. In these experiment we used high concentrations of the MPS1 (NMS-P715) and Aurora B (AZD1152) inhibitors (Supplementary Figure 1). When analysing asynchronous cells, 1.5 μM NMS-P715 caused a dramatic 5-fold reduction in mitotic timing, whilst 0.5 μM AZD1152 almost doubled the mitotic timing (Figure 1A); thus, Aurora B inhibitor-treated cells spend 10 times longer in mitosis than MPS1 inhibitor treated cells. Next, we assessed the ability of the inhibitors to override a nocodazole and taxol-induced SAC. Importantly, both nocodazole and taxol arrested the cells in mitosis for ∼19 hours, suggesting equivalent effective SAC activation (Figure 1A). However, when simultaneously treated with NMS-P715, cells were unable to establish a mitotic arrest in either nocodazole or taxol (Figure 1A). By contrast, when treated with AZD1152 and nocodazole, the cells still produced a robust SAC, arresting for ∼15 hours. However, in taxol, this mitotic arrest was greatly attenuated, although cells still initially arrested in mitosis for ∼5 hours. These data suggest that following Aurora B inhibition, the SAC is still established in cells, but not maintained.

We next compared the effects MPS1 and Aurora B inhibitors on overriding a pre-established nocodazole or taxol arrest. As expected, MPS1 inhibition was able to rapidly override both a nocodazole and taxol-induced arrest (Figure 1B), whilst 0.5 μM AZD1152 could only override a taxol-induced arrest (Figure 1B). Even at 1 μM, AZD1152 did not cause significant override of a nocodazole arrest (Supplementary Figure 2). These findings were confirmed looking at the formation of the MCC following immunoprecipitation of CDC20. In both nocodazole and taxol we observed strong binding of BUBR1, BUB3 and MAD2 to CDC20, in comparison to asynchronous cells (Figure 1C). When treated with NMS-P715, the binding of BUBR1, BUB3 and MAD2 to CDC20 were greatly reduced in nocodazole and taxol-arrested cells. However, AZD1152 had little effect on MCC formation in nocodazole, but reduced BUBR1, BUB3 and MAD2 binding in taxol. In summary, these data suggest that MPS1 activity is absolutely essential in order to both establish and maintain the SAC in mitosis. However, while Aurora B activity is dispensable for the initial establishment of the SAC, it is required to maintain the SAC signal, at least in the presence of the mitotic spindle.

### The recruitment of SAC proteins to the unattached kinetochore is not affected by Aurora B inhibition

Having shown that Aurora B inhibition only affects the SAC and MCC in the presence of taxol, we investigated the roles of MPS1 and Aurora B in protein recruitment to the kinetochore. Cells were arrested at metaphase using the proteasome inhibitor MG132, then treated with nocodazole and MG132 to initiate maximum re-recruitment of proteins to the unattached kinetochore [5]. Using NMS-P715, the recruitment of HEC1, KNL1, ZWINT1, CENPE and pCENP-A were unaffected, but BUB1, BUBR1, ZW10, CDC20, SPINDLY, MAD1 and MAD2 were all reduced (Supplementary Figure 3). Conversely, the recruitment of MPS1 doubled, despite the loss of the auto-phosphorylated T33/S37 signal, consistent with previous reports [5, 6, 23]. When arrested in taxol and MG132, NMS-P715 treatment showed similar results, with the exception of CENP-E that was now also reduced (Supplementary Figure 4).

**Figure 2:**
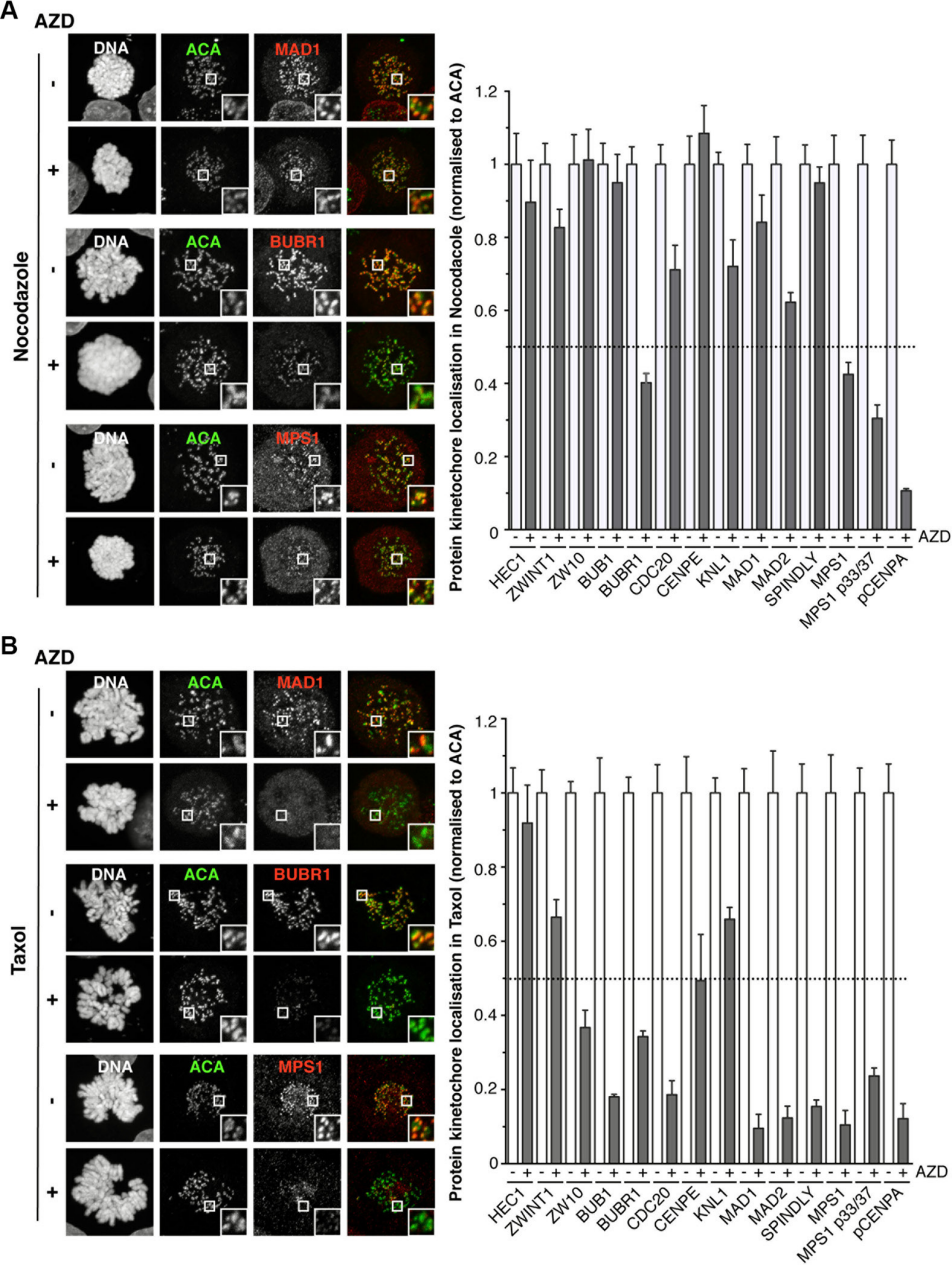
The effects of Aurora B inhibition on the localisation of proteins to the kinetochore (**A**) Immunofluorescence images of HeLa cells, showing the localisation of kinetochore proteins when arrested in nocodazole, in the absence or presence of 0.5 μM AZD1152. The white boxes are enlarged to highlight kinetochores. Bar graph quantifying pixel intensities at kinetochores normalized to ACA, are shown. The mean value from 7 cells +/– SEM are shown. (**B**) Immunofluorescence images of HeLa cells, showing the localisation of kinetochore proteins when arrested in taxol, in the absence or presence of 0.5 μM AZD1152. The white boxes are enlarged to highlight kinetochores. Bar graph quantifying pixel intensities at kinetochores normalized to ACA, are shown. The mean value from 7 cells +/– SEM are shown.

When looking at the effect of AZD1152 on the kinetochore re-recruitment of proteins in nocodazole, HEC1, ZWINT1, KNL1, BUB1, CDC20, CENP-E, MAD1, MAD2 and SPINDLY recruitment were largely unaffected, all remaining above 60% (Figure 2A and Supplementary Figure 5A). However, both BUBR1 and MPS1 kinetochore localisation were reduced to ∼40%, although still clearly visible. Importantly, CENP-A phosphorylation was completely lost, consistent with potent Aurora B inhibition (Figure 2A and Supplementary Figure 5A). However, when arrested in taxol AZD1152-treatemnt caused stark differences in protein localisation: BUB1, BUBR1, CENP-E, ZW10, SPINDLY, MAD1, MAD2, CDC20, and MPS1 were all now severely reduced at the kinetochore (Figure 2B and Supplementary Figure 5B), while HEC1, ZWINT1 and KNL1 remained localised (Figure 2B and Supplementary Figure 5B). In conclusion, these data demonstrate that Aurora B activity is dispensable for the recruitment of the majority of SAC proteins to unattached kinetochores, with the exception of BUBR1 and MPS1, but is required when treated with taxol, which allows attachment but with low tension. In line with this observation, in an asynchronous population of cells treated with AZD1152, despite potent Aurora B inhibition, BUB1 localisation is normal in both prophase and early prometaphase cells, but severely reduced in cells that appear to be later in mitosis (Supplementary Figure 6).

### Loss of Aurora B activity causes pre-mature removal of SAC proteins from the kinetochore

**Figure 3:**
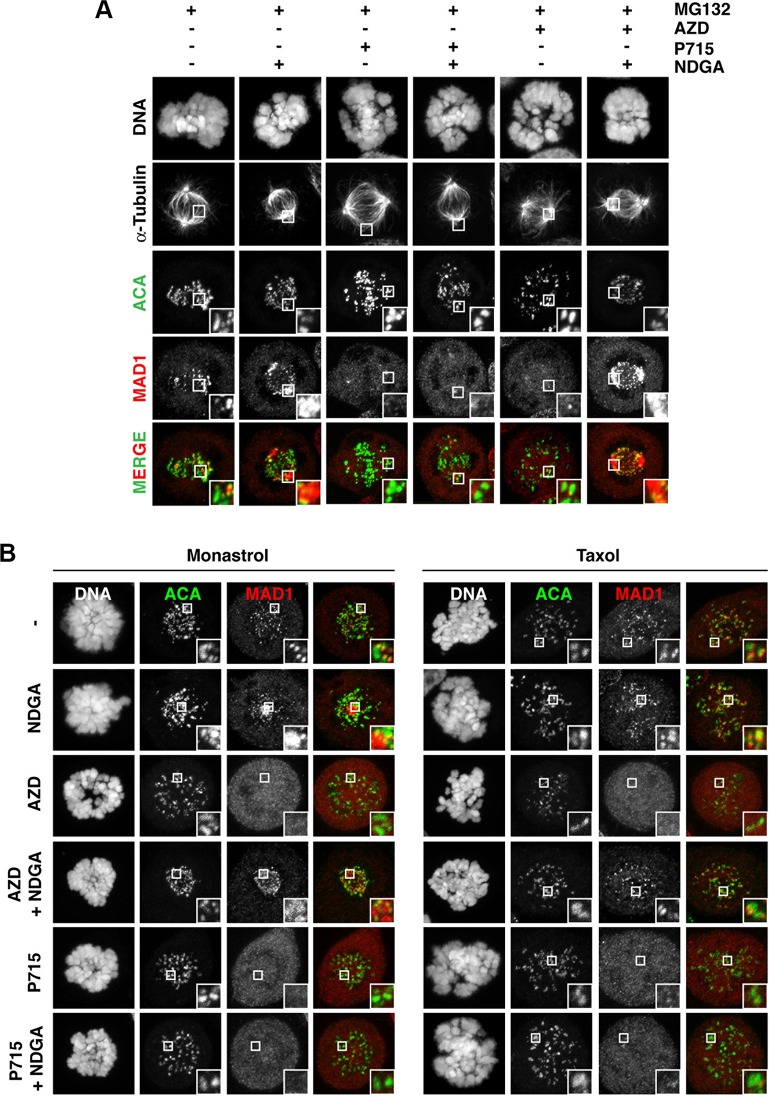
Aurora B inhibition causes the premature removal of SAC proteins from the kinetochore (**A**–**B**) Immunofluorescence images of HeLa cells, showing the localisation of MAD1, when treated with the indicated drugs for one hour. For (**B**), cells were first treated with monastrol or taxol for 1 hour, prior to addition of the other drugs. MG132 was added in all conditions. The white boxes are enlarged to highlight kinetochores and/or centrosome staining. AZD; 0.5 μM AZD1152, P715; 1.5 μM NMS-P715.

We examined two hypotheses to explain the loss of protein kinetochore localisation in taxol when treated with AZD1152: a) Aurora B activity is required for protein recruitment in response to kinetochores attachment to microtubules under low tension, or b) Aurora B prevents protein removal from the kinetochore. To investigate these theories, we analysed dynein-mediated stripping of SAC proteins using Nordihydroguaiaretic acid (NDGA), which enhances the interaction between dynein/dynactin and its cargo, allowing us to visualise protein stripping [24, 25]. Note that NDGA is not a dynein inhibitor. First, we confirmed the effect of NDGA on the localisation of MAD1; NDGA treatment caused dynein-bound MAD1 to strongly accumulated at the centrosome (Figure 3A), indicating inhibition of MAD1 release from dynein, consistent with previous observations [25]. We hypothesised that the inhibition of MAD1 kinetochore recruitment should prevent its centrosomal accumulation following NDGA treatment. In agreement with this, when treated with NMS-P715, no kinetochore or centrosomal MAD1 accumulation was evident (Figure 3A). In stark contrast, despite the complete loss of MAD1 kinetochore localisation in AZD1152-treated cells, co–treatment with NDGA caused a strong accumulation of dynein-bound MAD1 at both the kinetochore and the centrosome/spindle (Figure 3A). These data strongly suggest that the low levels of MAD1 observed following Aurora B inhibition is due to its removal from the kinetochore, at least partially via dynein-mediated stripping, which NDGA treatment allows us to visualise. The appearance of MAD1 following NDGA treatment suggests MAD1 is still recruited to the kinetochore. Similar to MAD1, the kinetochore and centrosomal accumulation of GFP-MPS1, CENP-E, SPINDLY, hDIC and CDC20 were seen following co-treatment of NDGA with AZD1152 (Supplementary Figure 7), but only kinetochore localisation was seen for BUB1, BUBR1 and CENP-F, possibly due to the antibody used, experimental timing, or perhaps dynein does not strip these SAC proteins fully to the centrosome (Supplementary Figure 7).

Next, we aimed to address whether we would observe similar results in taxol or the Eg5 inhibitor monastrol, which both allow KT-MT interactions, when using NDGA. In monastrol-treated cells, NDGA treatment caused a striking kinetochore and centrosomal accumulation of dynein-bound MAD1 (Figure 3B); kinetochore accumulation of MAD1 occurred within 30 mins, whilst centrosomal accumulation was not seen until 60 mins of NDGA treatment, thus kinetochore accumulation, followed by stripping upon NDGA treatment takes time (Supplementary Figure 8). NDGA treatment also allowed us to visualise the kinetochore and centrosome localization of dynein-bound MAD1 following treatment with AZD1152, but not NMS-P715 (Figure 3B). However, we never detected centrosomal accumulation of MAD1 in taxol following NDGA treatment, (Figure 3B), perhaps due to the loss of normal microtubule dynamics, although MAD1 kinetochore localisation was still restored in AZD1152 co-treated cells. Since dynein must bind to MAD1 at the kinetochores this may be unsurprising. This may also suggest that in taxol, mechanisms other than dynein-mediated stripping are also important for removal of the SAC proteins. In line with this observation, and consistent with previous report [26, 27], RNAi of either dynein heavy chain or SPINDLY cannot prevent the override of a taxol-induced arrest by AZD1152, despite delaying checkpoint silencing in asynchronous cells (Supplementary Figure 9A–9B). NDGA treatment also could not prevent the override induced by AZD1152 (Supplementary Figure 9C). However, both SPINDLY and DHC RNAi did prevent the centrosomal accumulation of MAD1 following NDGA treatment, in both the absence and presence of AZD1152, suggesting it is a dynein-mediated process (Supplementary Figure 10).

### Constitutive MPS1 localisation to the kinetochore cannot prevent override of the SAC induced by Aurora B inhibition

**Figure 4:**
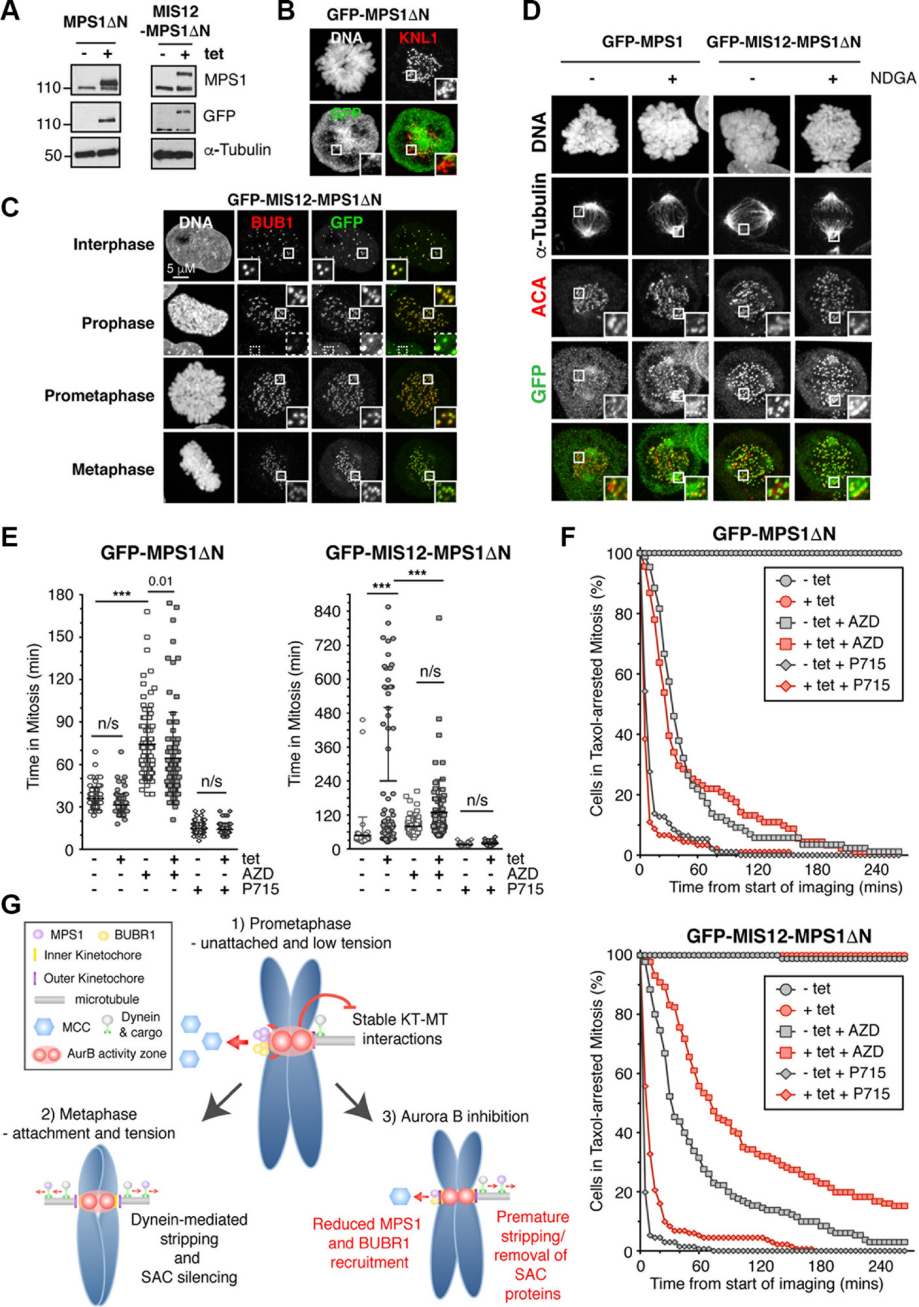
Aurora B inhibition can override the SAC induced by constitutive kinetochore localisation of MPS1 (**A**) Immunoblot showing the tetracycline (tet) inducible expression of GFP-MPS1ΔN and GFP-MIS12-MPS1ΔN in HeLa Flp-In T-Rex cells. (**B**–**C**) Immunofluorescence images showing the localisation of (B) GFP-MPS1ΔN and (C) GFP-MIS12-MPS1ΔN. The white boxes are enlarged to highlight the localisation. The dotted white box (C) is to highlight the reduced BUB1 localisation in interphase cells, as compared to during mitosis. (**D**) Immunofluorescence images showing the localisation of GFP-MPS1 and GFP-MIS12-MPS1ΔN following 1 hour treatment with NDGA. (**E**) Scattered dot plots showing the time spent in mitosis of asynchronous HeLa Flp-In T-Rex cells (stably expressing Histone H2B-mCherry) when induced (+tet) to express GFP-MPS1ΔN (left) and GFP-MIS12-MPS1ΔN (right), in the absence and presence of AZD1152 (AZD) and NMS-P715 (P715). The results were analysed by One-way ANOVA with ^***^ indicating *p* < 0.0001 and n/s = not significant. *N* = > 58 cells per condition. (**F**) Line graphs showing the mitotic exit of cells, pre-treated for 18 hours in taxol, then treated with 0.5 μM AZD1152 (AZD, squares) and 1.5 μM NMS-P715 (P715, diamonds) at 0 mins, in the absence (-tet, grey) and presence (+tet, red) of GFP-MPS1ΔN (top) and GFP-MIS12-MPS1ΔN (bottom). *N* = > 63 cells per condition. (**G**) Model illustrating the role of Aurora B in the SAC. Aurora B localizes to the centromere and can phosphorylate substrates within an Aurora B activity zone. In a normal mitosis (1) Aurora B promotes MPS1 and BUBR1 localisation to the outer kinetochore to enhance formation of the MCC. Simultaneously, it inhibits dynein-mediated stripping of the SAC proteins from kinetochores not under tension. When kinetochores are correctly attached to microtubules and under tension (2), the centromere and kinetochores are stretched, the outer kinetochore is removed from the Aurora B activity zone and SAC proteins are stripped. When Aurora B is inhibited (3), there is reduced MPS1 and BUBR1 kinetochore recruitment and SAC proteins are stripped from the kinetochore when attached to microtubules, despite the lack of tension.

Our results have shown a clear role for Aurora B in preventing the premature removal of SAC proteins from the kinetochore after KT-MT interactions. Since Aurora B inhibition also causes the stripping of MPS1, we questioned whether preventing MPS1 removal would prevent checkpoint inactivation by Aurora B inhibition. To this end, we expressed a GFP-MIS12-MPS1ΔN fusion protein in HeLa Flp-In T-Rex cells, which constitutively binds to the kinetochore. In addition, the first 192 amino acids of MPS1 were missing, removing the Aurora B regulated region [8]. GFP-MIS12-MPS1ΔN and GFP-MPS1ΔN both expressed to similar levels upon tetracycline induction (Figure 4A), however, whilst little if any kinetochore localisation of GFP-MPS1ΔN was detected (Figure 4B), GFP-MIS12-MPS1ΔN strongly localised to the kinetochore in interphase and mitosis (Figure 4C), consistent with previous reports [28]. This localisation was sufficient to recruit BUB1 to the kinetochore in interphase, although at much lower levels than detected in mitosis (Figure 4C). However, GFP-MIS12-MPS1ΔN was not sufficient to recruit other SAC proteins in interphase or prophase (Supplementary Figure 11), consistent with reports in *S. pombe* [29]. Importantly, GFP-MIS12-MPS1ΔN was also largely resistant to dynein-mediated stripping, with minimal centrosomal accumulation following NDGA treatment (Figure 4D).

To address whether GFP-MIS12-MPS1ΔN prevented checkpoint override induced by Aurora B inhibition, we analysed the mitotic timing of asynchronous cells. Expression of GFP-MIS12-MPS1ΔN considerably prolonged the length of time cells spent in mitosis (Figure 4E); one population of cells arrested at metaphase for ∼71 mins before entering anaphase, whilst a second population of cells remained in metaphase for ∼140 mins, followed by cohesion fatigue and mitotic arrest (Figure 4E). Importantly, GFP-MIS12-MPS1ΔN expression could not establish a SAC signal when treated with NMS-P715 (Figure 4E), whilst treatment with AZD1152 abolished the prolonged arrest caused by GFP-MIS12-MPS1ΔN expression; no cells entered cohesion fatigue (Figure 4E). These data suggest that Aurora B inhibition reduced the persistent SAC induced by forced MPS1 kinetochore localisation. To further confirm this, we repeated the experiment using cells pre-arrested in mitosis with taxol (Figure 4F). Under these conditions, GFP-MIS12-MPS1ΔN caused a mild delay in mitotic exit following AZD1152 treatment, suggesting that a hyperactive SAC delayed AZD1152-mediated override. Likewise, a similar fold delay was also seen with NMS-P715 (Figure 4F). Taken together, these data suggest that Aurora B inhibition reduces the persistent SAC signal induced by forced MPS1 kinetochore localisation and consistent with the idea that Aurora B prevents premature SAC protein removal following KT-MT interactions. In agreement with this, despite the persistent localisation of GFP-MIS12-MPS1ΔN in taxol-arrested cells treated with AZD1152, the kinetochore localisation of BUB1, MAD1 and MAD2 are still reduced (Supplementary Figure 11B). In conclusion, our data suggests Aurora B has a dual role in the SAC (modeled in Figure 4G); 1) Aurora B activity enhances MPS1 and BUBR1 recruitment to activate the SAC (Figure 2A), and 2) it prevents the premature removal of SAC proteins from the kinetochore, by preventing the formation of stable KT-MT interactions (Figure 3). Thus, in an unperturbed metaphase, when kinetochores are attached, stable and under tension, MPS1 kinetochore recruitment is reduced and its removal via dynein-mediated stripping (among other mechanisms) is increased, along with the removal of other SAC proteins, therefore promoting SAC silencing (Figure 4G).

### MPS1 and Aurora B act synergistically in maintaining the SAC in a microtubule dependent manner

**Figure 5:**
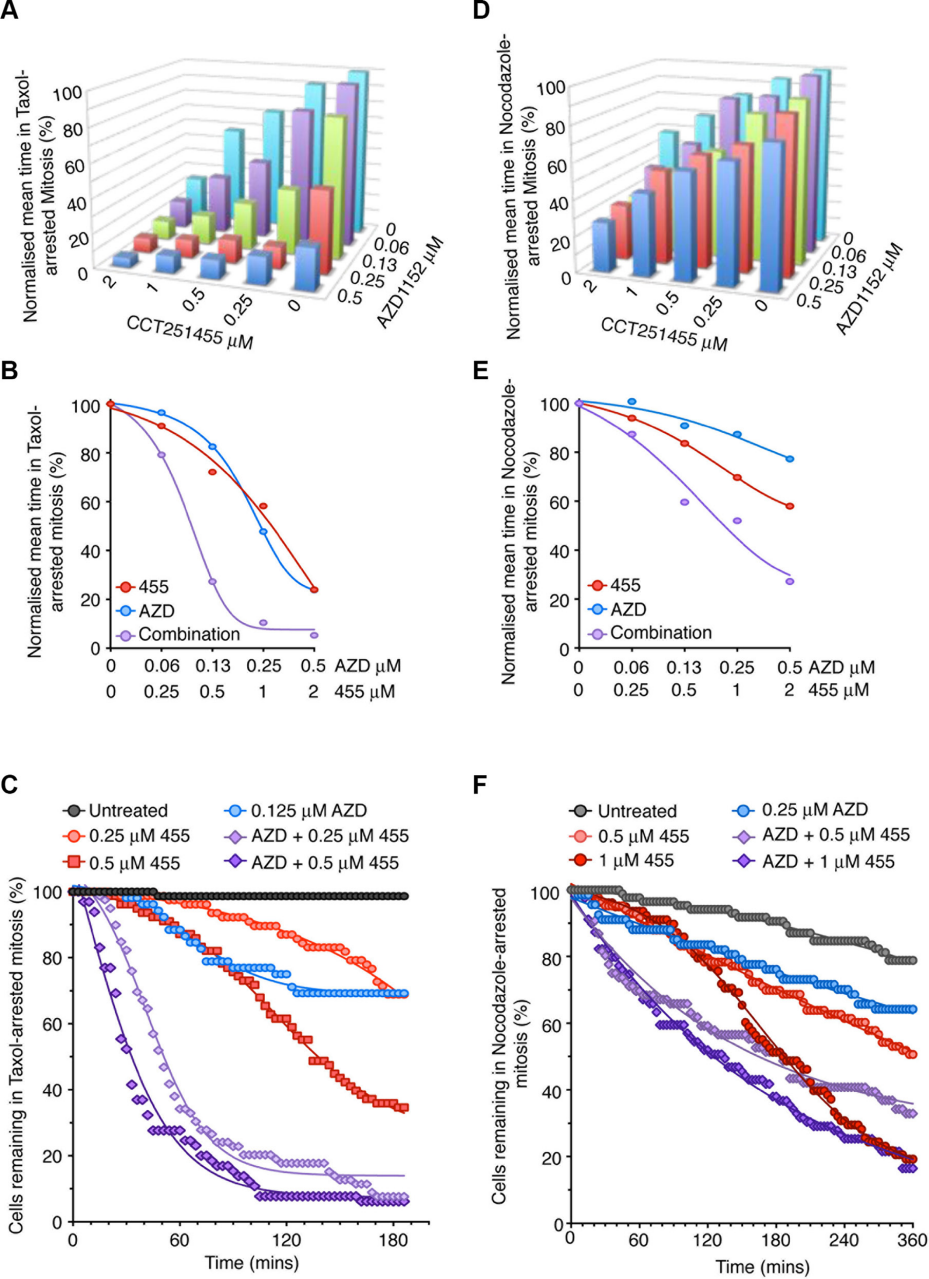
MPS1 and Aurora B inhibitors synergise to override a taxol-induced-arrest (**A**–**B**) Bar graph (A) and line graph (B) showing the normalized average time cells remained in taxol-induced arrest, following 3 hours treatment with CCT251455 and AZD1152. *N* = > 52 cells per conditions. (**C**) Line graph showing the exit from a taxol-induced mitotic arrest of individual cells, following treatment with CCT251455 and AZD1152. (**D**–**E**) Bar graph (D) and line graph (E) showing the normalized average time cells remained in nocodazole-induced arrest, following 5 hours treatment with CCT251455 and AZD1152. *N* = > 52 cells per conditions. (**F**) Line graph showing the exit from a nocodazole-induced mitotic arrest of individual cells, following treatment with CCT251455 and AZD1152.

It has previously been suggested that MPS1 and Aurora B inhibitors act synergistically to prevent a SAC response when treated with nocodazole [11, 21]. However, we show Aurora B inhibition could not override a pre-established nocodazole-arrest, despite the small reduction in mitotic arrest when treating prior to mitotic entry (Figure 1). These data are consistent with previous reports that Aurora B inhibition delays, not prevents, initial MPS1 recruitment and establishment of the SAC [11]. Thus, we examined the potential synergy between MPS1 (CCT251455), a potent, selective and orally bioavailable MPS1 inhibitor [30] and Aurora B (AZD1152) inhibitors in a pre-established SAC by time-lapse microscopy (Figure 5A). When arrested in taxol, both CCT251455 and AZD1152 individually stimulated mitotic exit, however, co-treatment showed clear synergy; 125 nM AZD1152 and 500 nM CCT251455 increased SAC override from ∼20% individually, to 80% in combination (Figure 5A–5B) Indeed, analysis using Macsynergy™II [31] indicated a high synergy score (Supplementary Figure 12A). This synergy was more striking when plotting the mitotic exit of cells over time; 125 nM AZD1152 or 500 nM CCT251455 alone caused ∼10% of cells to exit mitosis by 60 mins, but in combination ∼80% of the cells had exited the taxol-induced arrest (Figure 5C). By contrast, when cell were arrested overnight in nocodazole, this synergy was greatly attenuated, consistent with the Aurora B inhibitor having little effect on checkpoint override in nocodazole-arrested cells (Figure 7D–7F and Supplementary Figure 12B). These results suggest that the high synergy seen between MPS1 and Aurora B inhibitors in overriding the SAC is largely dependent on the microtubule spindle.

### Different fates of cells treated with MPS1 and Aurora B inhibitors

Since MPS1 and Aurora B play distinct roles in the SAC, we wanted to determine whether this affected cell fate. To this end, we analysed HeLa Fucci cells by time-lapse microscopy over 72 hours, following treatment with MPS1 or Aurora B inhibitors. HeLa Fucci cells express different fluorescent markers in G1 and G2, thereby allowing us to identify when cell-death occurs.

**Figure 6:**
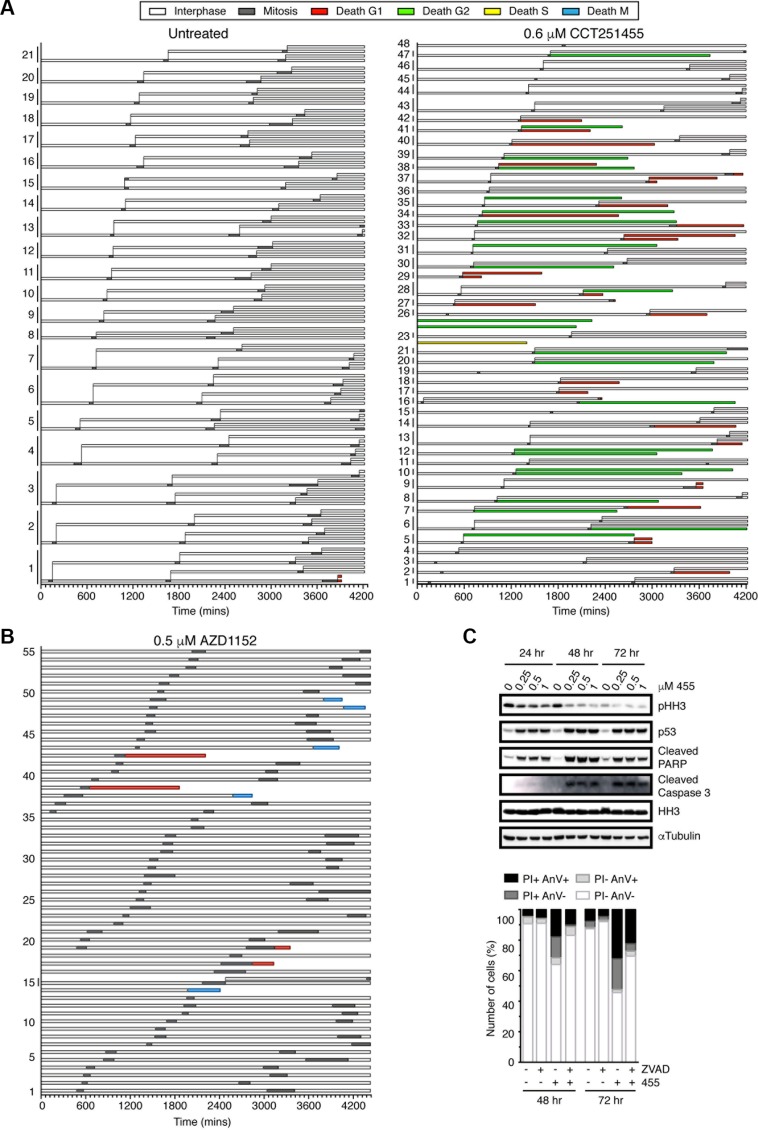
The cell fate profiles are different in response to MPS1 and Aurora B inhibition (**A**–**B**) Bar graphs representing the fate of untreated, CCT251455 and AZD1152-treated HeLa Fucci cells over 72 hours. The length of the bar represents the time spent in that cell cycle phase. Progeny are grouped according to their parent. The cells were imaged every 10 mins. (**C**) Immunoblot (top) and a bar graph quantifying annexin V and PI staining (Bottom), showing the induction of apoptosis in HCT116 cells treated with CCT251455 over 72 hours.

Consistent with MPS1 inhibition, CCT251455-treated cells spent less time in mitosis compared to untreated cells (Figure 6A), however, of the 113 cells analysed only 54 died (47.7%): 29 in G1, 1 in S-phase and 24 in G2 (Figure 6A). Interestingly, the cells that died in G2 mostly did so after one aberrant mitosis (21 of 24 cells), whereas death in G1 typically occurred after two aberrant mitoses (19 of 29 cells). Cell death was time-dependent, occurring predominantly after 48 hours, but was not strictly dependent on progressing through multiple mitoses, since 57% of cells died after one aberrant mitosis. In agreement with these data, cleaved caspase 3, cleaved PARP and p53 induction was largely observed after 48 hours CCT251455 treatment in HCT116 cells (Figure 6C). Furthermore, Annexin V and PI staining of apoptotic cells increased to ∼50% between 48–72 hours, which was partially reduced by the pan-caspase inhibitor Z-VAD-FMK (Figure 6C). We also determined that inhibitor-treated cells spent longer in interphase between the first and second aberrant mitoses (Figure 6A). These data suggest that MPS1 inhibition causes a cell cycle arrest, as well as interphase death in both G1 and G2 phases of the cell cycle following an aberrant mitosis (both a cytostatic and cytotoxic effect), thereby reducing the cell viability to ∼25% of control cells.

When treated with AZD1152, all but 1 cell underwent polyploidisation, due to cytokinesis failure (Figure 6B). However, in contrast to MPS1-inhibitor treated cells, no interphase delay was detected and only 9 of the 56 cells died; 5 in mitosis and 4 in G1. Thus, Aurora B inhibition appeared to be much less efficient at inducing cell death than MPS1 inhibitors, at least after shot term treatment. Furthermore, p53 deficient HCT116 cells were largely unresponsive to AZD1152 in a 4-day cell viability assay, causing only ∼20% cell death compared to the 60% cell death in wild-type cells, or 60% following CCT251455 treatment, despite obvious polyploidisation and chromosome segregation defects, seen by time-lapse microscopy (Supplementary Figure 13). This suggests that the immediate response of cells to Aurora B inhibitors is markedly different to MPS1 inhibitors, being more greatly affected by the p53 pathway.

### MPS1 and Aurora B synergise in killing cancer cells though override of the SAC

**Figure 7:**
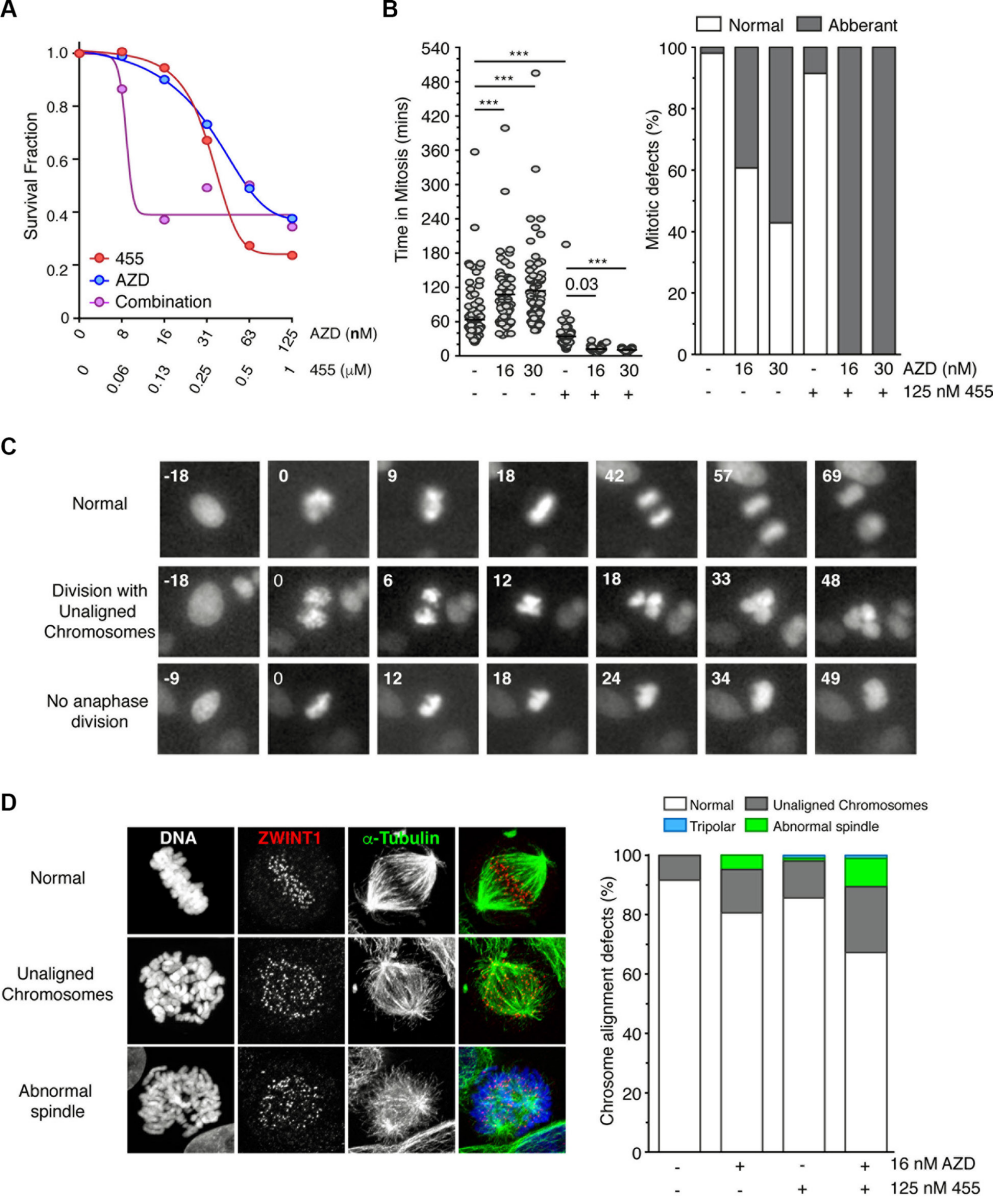
MPS1 and Aurora B inhibitors synergise in killing cells through override of the SAC (**A**) Line graph showing the cell viability of HeLa cells in response to CCT251455 and AZD1152, alone and in combination. The mean of three experiments is shown. (B - left) Scattered dot plots showing the time spent in mitosis of asynchronous HeLa cells (stably expressing Histone H2B-mCherry) in the absence and presence of CCT251455 and/or AZD1152. (B – right) Bar graph quantifying the chromosome segregation defects. *N* = > 46 cells per condition and analysed by One-way ANOVA, with ^***^ indicating *p* < 0.0001. (**C**) Representative time-lapse images from (**B**) showing a normal mitosis and the chromosome segregation defects following treatment with 30 nM AZD1152 and 125 nM CCT251455. (**D**) Representative images (left) and bar graph quantification (right) of the chromosome alignment defects in HeLa cells treated for 90 mins with MG132, AZD1152 and/or CCT251455, then fixed for immunofluorescence. *N* = > 100 mitotic cells counted per condition. AZD = AZD1152; 455 = CCT251455.

Thus far, our data has shown that MPS1 and Aurora B inhibition synergise in overriding a taxol, but not a nocodazole-induced mitotic arrest. Furthermore, MPS1 is comparatively more proficient in inducing cell death. Consequently, we wanted to address whether MPS1 and Aurora B inhibition would also result in the synergistic killing of cancer cells. Indeed we found that MPS1 and Aurora B inhibitors acted synergistically in killing asynchronous HeLa cells; 16 nM AZD1152 and 125 nM CCT251455 caused a maximum decrease in cell viability, whilst causing a < 10% reduction individually (Figure 7A). Analysis by Macsynergy™II showed this interaction to be highly synergistic (Supplementary Figure 12C). To address the mechanism of this synergy we analysed the mitotic timing of HeLa cells by time-lapse microscopy. AZD1152 caused a significant increase in mitotic timing (from 60 to 100 mins), accompanied by increased defects in chromosome segregation (Figure 7B–7C). Conversely, 125 nM CCT251455 reduced mitotic timing to ∼30 mins, although only a minor increase in segregation defects were detected (Figure 7B). The combination of both drugs caused a dramatic decrease in mitotic timing (< 20 mins) with 100% of cells having abnormal mitoses; cells either divided with unaligned chromosomes, or the chromosomes decondensed *en masse* without division (Figure 7C). These results were recapitulated using the pan-Aurora inhibitor CCT241736 [32] (Supplementary Figure 14). To address the contribution of defects in chromosome alignment, independent of SAC activity, we arrested cells in mitosis with MG132 (Figure 7D). However, only marginally alignment defects were detected and no synergy was seen, suggesting that the synergistic increase in cell death is predominantly caused through override of the SAC.

**Figure 8:**
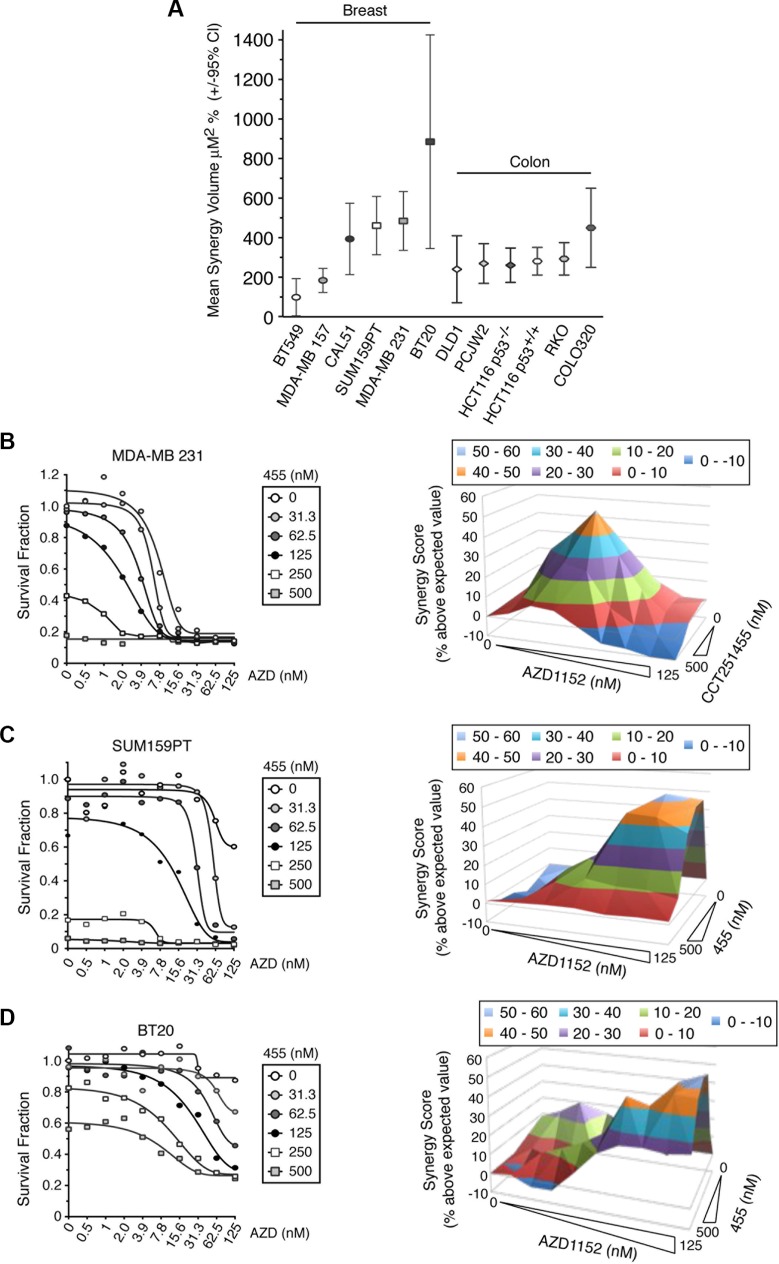
MPS1 and Aurora B inhibitors synergistically kill cancer cells unresponsive to Aurora B inhibitors alone (**A**) A graph showing the mean synergy volume of cells treated with AZD1152 and CCT251455. The graph represents the mean of 3 experiments +/– 95% confidence interval. (**B**–**D**) Line graphs (left) and synergy 3-D plots (right) showing the synergistic killing of MDA-MB 231 (B), SUM159PT (C) and BT20 (D) cells when treated with AZD1152 and CCT251455. The mean of 3 experiments is shown. AZD = AZD1152; 455 = CCT251455.

Finally, to show whether MPS1 and Aurora B inhibitors could synergistically kill other cancer cell lines, we tested a panel of six basal-type breast cancer cell lines and six colon cancer cell lines (Figure 8 and Supplementary Figure 15). All the cell lines examined, with the exception of BT549, showed a strong synergistic response to MPS1 and Aurora B inhibitors, with a high synergy volume using Macsynergy™II analysis (Figure 8A). Typically, cells that responded to both drugs produced a “synergy pyramid”, such as for MDA-MB-231 (Figure 8B). However, of particular interest, whilst BT20, SUM159PT and MDA-MB 157 cells showed a minimal response to AZD1152 alone (between 10–40%), co-treatment with low doses of CCT251455 caused a dramatic increase in cell death, causing non-pyramid synergy plots (Figure 8C–8D). For BT20 cells, which also did not respond fully to MPS1 inhibition, a very high synergy score was seen, since the combination of inhibitors resulted in higher cell death than either inhibitor alone (Figure 8D). These results suggest that MPS1 inhibitors may sensitise cell lines that were otherwise unresponsive to Aurora B inhibitors (Figure 8C–8D) and the combination of MPS1 and Aurora B inhibitors may have potential use as an anti-cancer therapy in the clinic.

## DISCUSSION

In this current study we have extensively characterised the effects of MPS1 and Aurora B inhibition on the SAC and present data to demonstrate that the key role of Aurora B in the SAC is preventing premature kinetochore removal of SAC proteins following KT-MT interactions, independent of MPS1. We show that while Aurora B kinase activity enhances, but is not strictly essential, for MPS1 and BUBR1 kinetochore localisation, as previously suggested [11], it is largely dispensable for the recruitment of other SAC proteins and the establishment of the SAC. However, Aurora B activity is critical in maintaining the SAC signal, through preventing the premature removal of the kinetochore bound proteins, which normally is at least partially mediated by dynein-mediated stripping and is dependent on KT-MT interactions (modelled in Figure 4G). Consequently, MPS1 and Aurora B inhibitors strongly synergise in overriding the SAC, through simultaneously inhibiting SAC establishment caused by MPS1 inhibition and maintenance caused by Aurora B inhibition, thus inducing rapid cell death in cancer cell lines.

For many years it was not established why Aurora B inhibition can efficiently override a taxol-induced mitotic arrest, but not a nocodazole-arrested cells, although it had been hypothesised that stable KT-MT interactions may alone be sufficient for SAC silencing [19, 20]. It was recently suggested that this difference was due to the incomplete inhibition of Aurora B activity, since high doses of hesperadin could overcome this arrest [21]. However, even excessive concentrations of the Aurora B inhibitor AZD1152 were insufficient to override a nocodazole-induced SAC, suggesting the override seen with hesperadin was due to off-target effects [33, 34]. Once the checkpoint is established in nocodazole, our data shows that Aurora B activity is largely dispensable for SAC production, since, despite reduced MPS1 and BUBR1 kinetochore localisation, all other SAC proteins continue to be robustly recruited and the MCC formed. However, when treated with taxol, Aurora B is essential to prevent removal of the SAC proteins from the kinetochore. Using NDGA, which enhances the interaction between dynein/dynactin and its cargo [24, 25], we show that the loss of kinetochore localisation following Aurora B inhibition is due to the premature removal of proteins from kinetochores, which is at least partially mediated by dynein-mediated stripping in a normal mitosis. However, dynein-mediated stripping is not strictly essential for SAC silencing. Thus, it would appear that Aurora B has a dual role in regulating MPS1 kinetochore localisation and the SAC; firstly, it enhances the recruitment of MPS1 (and BUBR1) to rapidly establish the SAC, secondly, as KT-MT interactions are formed, Aurora B prevents the premature removal of MPS1 and other SAC proteins from tensionless kinetochores, likely through its role in error correction. This explains the high synergy between MPS1 and Aurora B inhibitors on the SAC in the presence of spindle microtubules, which is lost when cells are pre-arrested in nocodazole. Consistent with this idea, Aurora B inhibition can silence the checkpoint at metaphase arrest when MAD1 was constitutively tethered to kinetochores [22], as well as when MPS1 is tethered to the kinetochore and arrested in mitosis with taxol. Interestingly, during the preparation of this manuscript, back-to-back publications suggested that hyper-stable KT-MT interactions, generated by expressing a mutated HEC1 preventing its phosphorylation, are sufficient to enable premature SAC silencing [35, 36]. This further supports our own findings, since HEC1 is phosphorylated by Aurora B to regulate the affinity of KT-MT interactions [37, 38]. However, whether Aurora B has a more direct role in regulating SAC silencing mechanisms remains unknown and may warrant further investigation, since a number of spindle checkpoint-silencing mechanisms have been proposed in mammalian cells [39]. Recent work in budding yeast demonstrated that end-on microtubule attachment to the kinetochore physically separates MPS1, from its substrate Spc105 (KNL1), thus preventing continued MCC formation and allowing SAC silencing at metaphase [40]. It is interesting to speculate that this same mechanism may also physically separate Aurora B from substrates in the outer kinetochore, thereby allowing the generation of stable KT-MT interactions and SAC silencing. In support of this hypothesis, Aurora B function is dependent on its spatial separation from its kinetochore substrates [17, 37, 38, 41]. In fact, a MIS12-INCENP fusion protein is sufficient to cause a prolonged metaphase arrest, with only minor defect in chromosome alignment, suggesting SAC silencing may be prevented by Aurora B kinetochore localization [17]. Likewise an INCENP-mutant lacking its putative coiled-coil domain, is unable to sustain a taxol-induced SAC arrest [42], perhaps suggesting this domain is involved in SAC silencing, or confirms the dog-leash model, whereby the stretching of INCENP limits the area of Aurora B activity [43].

Anti-mitotic therapeutics such as taxanes or *vinca* alkaloids are widely used in the clinical treatment of cancer [44], causing cancer cell death through multipolarity, chromosome mis-segregation and aneuploidy [45]. Even so, the exact mechanism through which this triggers apoptosis remains elusive. A more recent report has suggested that generating aneuploidy, but not polyploidy, triggers p53 activation, the DNA damage response and causes proteotoxic stress, resulting in cell death [46]. Likewise, we show that MPS1 inhibition, which causes severe aneuploidy, efficiently kills cells in 72 hours, whilst Aurora B inhibition causes minimal cell death within this time-frame. Furthermore, AZD1152-mediated killing is almost completely suppressed in p53^-/-^ HCT116 cells up to 96 hours, whilst having minimal effect following MPS1 inhibition. Thus cancer cells may be much more tolerant to the generation of polyploidy. In fact, there is evidence that at least some cancer cells exposed to docetaxel not only escape its cytotoxic effects, but become more apoptosis resistant and malignant due to polyploidisation [47]. Furthermore, in yeast, tetraploid cells can undergo faster adaptation than their diploid counterpart, thus showing that polyploidy can accelerate evolutionary adaptation [48]. Perhaps this could explain the disappointing clinical efficacy seen with Aurora B inhibitors as a single agent [49, 50]. Our results suggests that combining Aurora B and MPS1 inhibitors not only synergise in killing cancer cell lines, through synergistic override of the SAC, but more importantly, sensitise breast cancer cell lines to cell death that otherwise do not respond to Aurora B inhibitors alone. This suggests the combination of Aurora B and MPS1 inhibitors may have great potential in the clinic as an anti-cancer therapy and deserves further exploration.

## MATERIALS AND METHODS

### Cell culture

HeLa Fucci cells were purchased form Life technologies. All other cell lines were purchased from ATCC. HeLa, Cal51, MDA-MB157, MDA-MB231, DLD1, PCJW2, HCT116, RKO, COLO320 cells were cultured in DMEM, supplemented with 10% FBS, 100 U/ml Penicillin and 100 μg/ml Streptomycin. BT20 and BT549 were cultured in RPMI media with 10% FBS, 100 U/ml Penicillin and 100 μg/ml Streptomycin. SUM159PT cells were cultured in 1:1 DMEM/HAM's F12, supplemented with 10% FBS, 100 U/ml Penicillin and 100 μg/ml Streptomycin, 5 μg/ml Insulin and 1 μg/ml Hydrocortisone. HeLa cell viability was assessed by CellTiterGlo after 96 hours, using a 96 well format (Promega). Breast and Colon cell line viability assays were assessed in a 384 well format after five days using Celigo S Imaging Cytometer (Nexcelom). Synergy was assessed using the “Macsynergy™II” spread sheet [31]. Tetracycline (Sigma) was used at a final concentration of 1 μg/ml, nocodazole (Sigma) at 200 ng/ml (0.66 μM), paclitaxel (Sigma) at 200 nM, MG132 (Sigma) at 20 μM, NDGA at 100 μM (Sigma) and monastrol at 40 μM (Sigma). CCT251455, CCT241736, NMS-P715 and AZD1152 were synthesised at the Institute of Cancer Research.

### Molecular cell biology

MPS1ΔN (missing the N-terminal 192 amino acids) was PCR amplified from MPS1 [23] and cloned into the modified pcDNA5/FRT/TO-GFP vector (courtesy of Prof. Stephen Taylor) using BamHI and NotI. MIS12 cDNA was amplified using ImProm-II Reverse transcription protocol (Promega) and cloned at the N-terminal of MPS1 using XhoI and BglII, introducing an Arg-Ser linker. Stably transfected, tetracycline-inducible HeLa Flp-In T-Rex cells were created as previously described [51].

### Immunofluorescence and time-lapse microscopy

Analysis by immunofluorescence and time-lapse microscopy were performed as previously described [23]. When using NDGA in immunofluorescence experiments, the cells were fixed for 20 mins in ice-cold methanol. Primary antibodies used were: α-tubulin (Sigma, T9026), ACA (ImmunoVision, HST-0100), BUB1 (Abcam, ab54893), BUBR1 (BD Biosciences, 612503), CENP-A pS7 (New England Biolabs, 2187S), CENP-E (Abcam, ab5093), MAD1 (Abcam, ab45286), MAD2 (Bethyl Laboratories Inc., A300-301A), CDC2020 (Millipore, MAB3775), hDIC (Abcam, ab23905), MPS1 (Millipore, 05–682), MPS1 pT33pS37 (Life Technologies, 44–1325G), GFP (Abcam, ab6556), HEC1 (Abcam, ab3613), Histone H3 pS10 (Millipore, 06–570), KNL1 (Bethyl Laboratories Inc., A300-805A), SPINDLY (Abnova, H00054908), ZW10 (Abcam, ab21580), ZWINT1 (Abcam, ab84367).

### Immunoprecipitation and immuoblotting

Cells were lysed at 4°C for 30 mins in lysis buffer: 30 mM Tris HCl, 150 nM NaCl, 2 mM EDTA, 10% Glycerol, 0.2% Triton X-100, with PhosSTOP (Roche) and Complete protease inhibitors (Roche). Lysates were incubated with anti-CDC20 antibody (Abcam, 26483) for 1 hr at room temperature, then incubated a further 15 mins with dynabeads (Life technologies). The beads were washed, bound proteins eluted using 0.2 M glycine [pH 2.5] and SDS loading buffer added prior to immunoblotting on NuPAGE Tris-Acetate gels (Life Technologies) as previously described [52]. Primary antibodies used were: α-tubulin (Sigma, T9026), BUB3 (BD Biosciences, 611731), BUBR1 (BD Biosciences, 612503), CDC20 (Millipore, MAB3775), GFP (Clonetech, 632381), Histone H3 (Abcam, ab1791), Histone H3 pS10 (Millipore, 06–570), MAD2 (Bethyl Laboratories Inc., A300-301A), MPS1 (Millipore, 05–682), MPS1 pT33pS37 (Life Technologies, 44–1325G), MPS1 pT676^30^, Cleaved PARP (Cell Signaling, 9541), Cleaved Caspase 3 (Cell Signaling, 9661), p53 (Thermo Fisher Scientific, MS-738-P).

### Apoptosis assay

Trypsinized cells were resuspended in binding buffer (10 mM HEPES [pH 7.4], 140 mM NaCl, 2.5 mM CaCl_2_), then incubated with 1.5 μM propidium Iodide (Fluka), 5 μl/ml Annexin V-FITC (Bender MedSystems) and analysed by flow cytometry.

## SUPPLEMENTARY MATERIALS FIGURES


