# Supplementary material for: Aurora B prevents premature removal of spindle assembly checkpoint proteins from the kinetochore: A key role for Aurora B in mitosis
Source: Oncotarget. 2016 Jul 18;9(28):19525–42. doi: 10.18632/oncotarget.10657 (PMC5929406; doi:10.18632/oncotarget.10657)
Supplement: Supplementary file 1 [file oncotarget-09-19525-s001.pdf]

Aurora B prevents premature removal of spindle assembly checkpoint proteins from the kinetochore: A key role for Aurora B in mitosis

Supplementary Materials

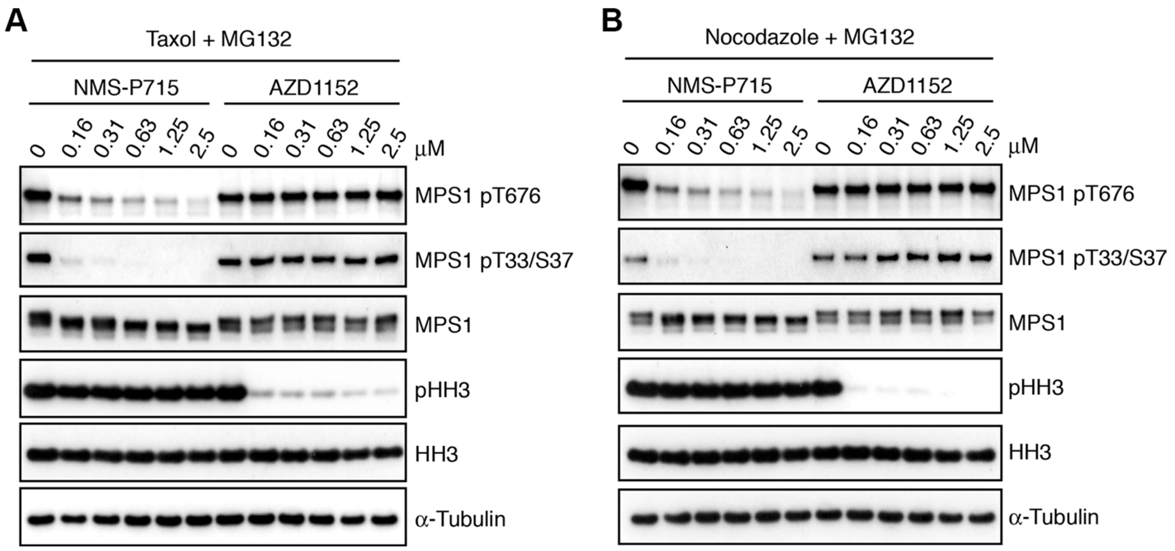

Supplementary Figure 1: Biomarker modulation following MPS1 and Aurora B inhibition in taxol and nocodazole. (**A**–**B**) Immunoblots showing the inhibition of MPS1 and Histone H3 phosphorylation by NMS-P715 and AZD1152 in cells arrested in taxol (A) and nocodazole (B).

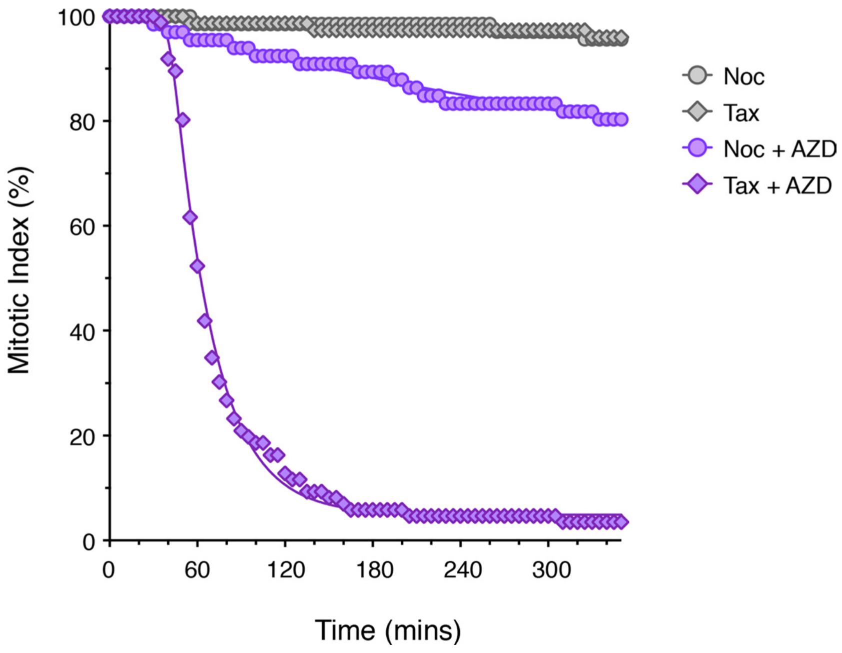

Supplementary Figure 2: High doses of AZD1152 cannot override the SAC when pre-arrested in nocodazole. Line graph showing the mitotic exit of cells, analysed by time-lapse, pre-arrested for 18 hours in nocodazole (noc) and taxol (tax), then treated with 1 µM AZD1152 (AZD) at 0 mins. *N* = > 66 cells per condition.

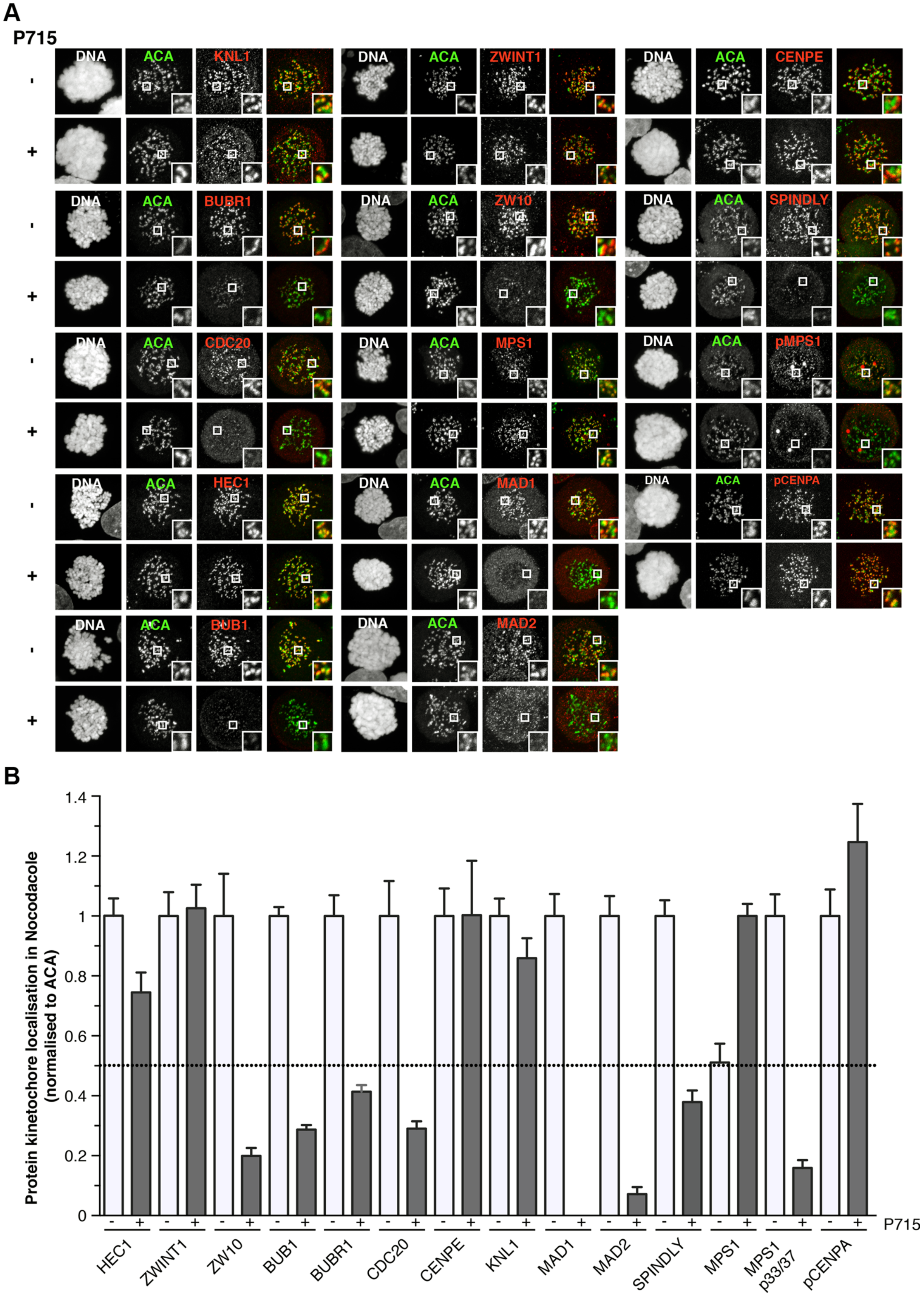

Supplementary Figure 3: The effect of MPS1 inhibition on the localisation of SAC proteins to the kinetochore in nocodazole. (**A**) Immunofluorescence images of HeLa cells, showing the localisation of kinetochore proteins when arrested in nocodazole, in the absence or presence of 1.5 μM NMS-P715. The white boxes are enlarged to highlight kinetochores. (**B**) Bar graph quantifying pixel intensities at kinetochores normalized to ACA, are shown. The mean values from 7 cells +/– SEM are shown.

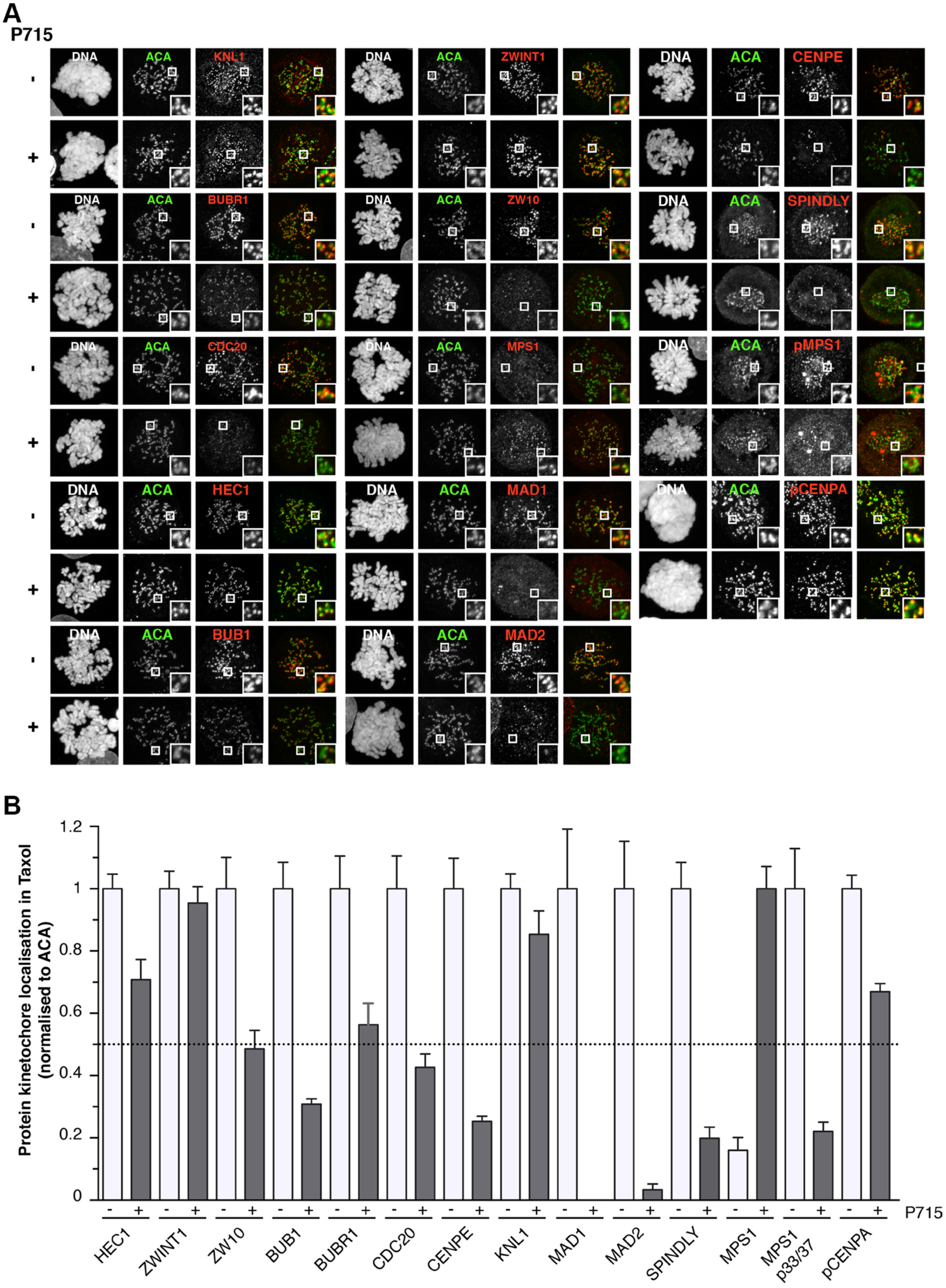

Supplementary Figure 4: The effect of MPS1 inhibition of the localisation of SAC proteins to the kinetochore in taxol. (**A**) Immunofluorescence images of HeLa cells, showing the localisation of kinetochore proteins when arrested in nocodazole, in the absence or presence of 1.5 µM NMS-P715. The white boxes are enlarged to highlight kinetochores. (**B**) Bar graph quantifying pixel intensities at kinetochores normalized to ACA, are shown. The mean values from 7 cells +/– SEM are shown.

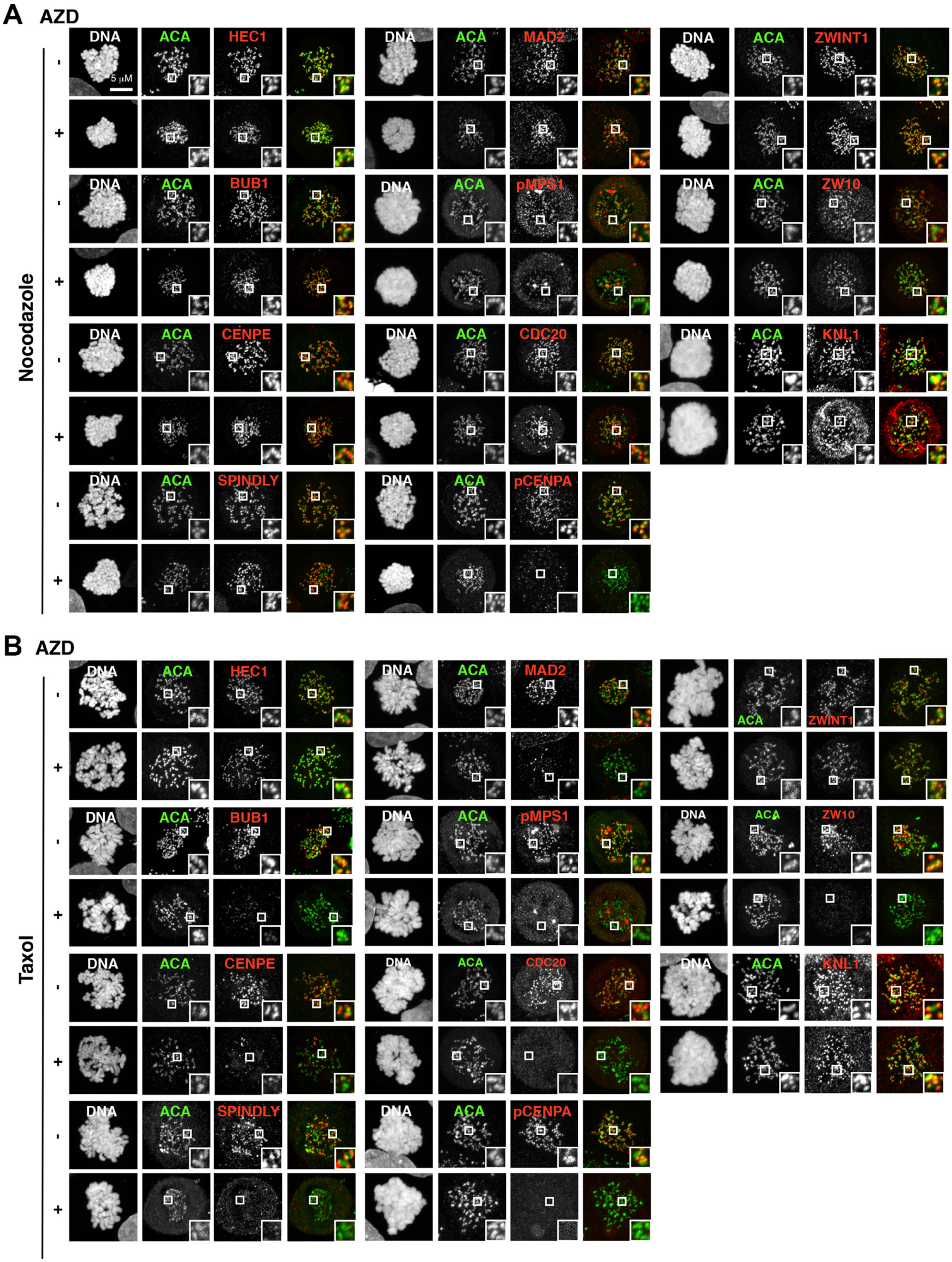

Supplementary Figure 5: The effect of Aurora B inhibition of the localisation of SAC proteins to the kinetochore. (**A**–**B**) Immunofluorescence images of HeLa cells, showing the localisation of kinetochore proteins when arrested in nocodazole (A), or taxol (B), in the absence or presence of 0.5 μM AZD1152. The white boxes are enlarged to highlight kinetochores.

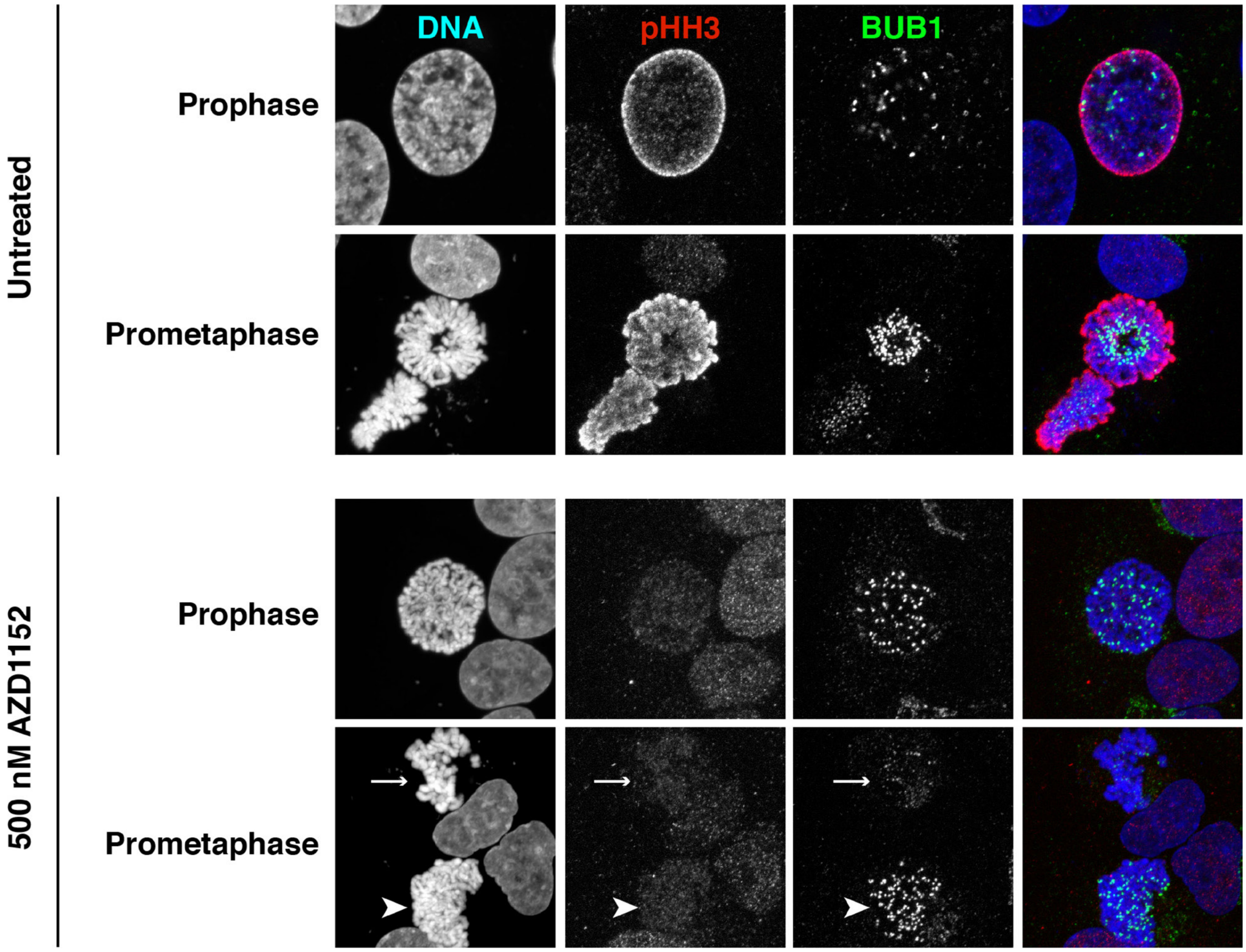

Supplementary Figure 6: The effect of Aurora B inhibition of the localisation of BUB1 to the kinetochore in an asynchronous population. Immunofluorescence images of HeLa cells, showing the localisation of BUB1 in asynchronous cells, in the absence or presence of AZD1152. The arrowhead highlights a cell in early prometaphase. The arrow shows a cell in late prometaphase with a visible metaphase plate forming.

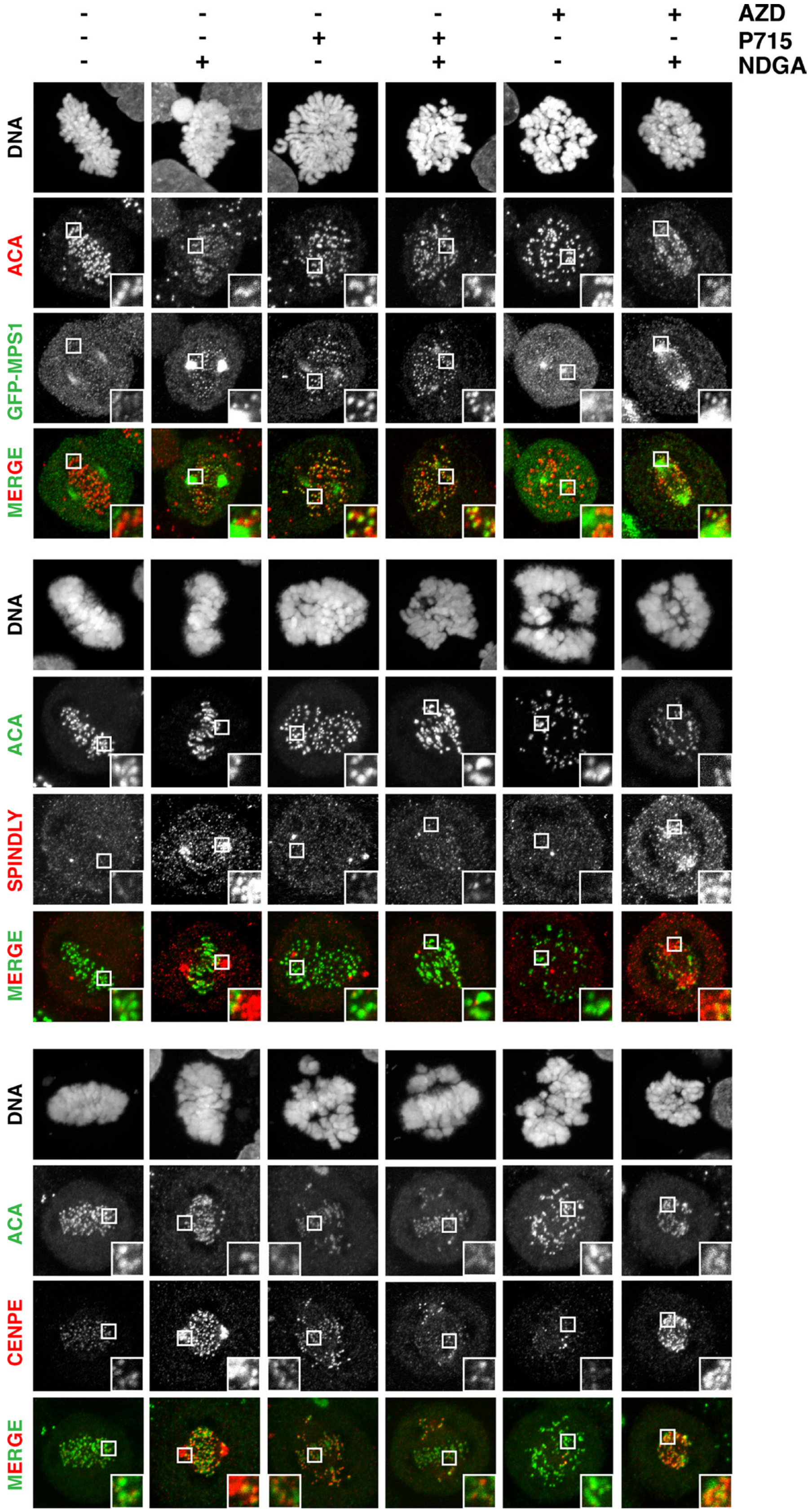

AZD
P715
NDGA
- - - - + +
- - + + - -
- + - + - +
DNA
ACA
GFP-MPS1
MERGE
DNA
ACA
SPINDLY
MERGE
DNA
ACA
CENPE
MERGE

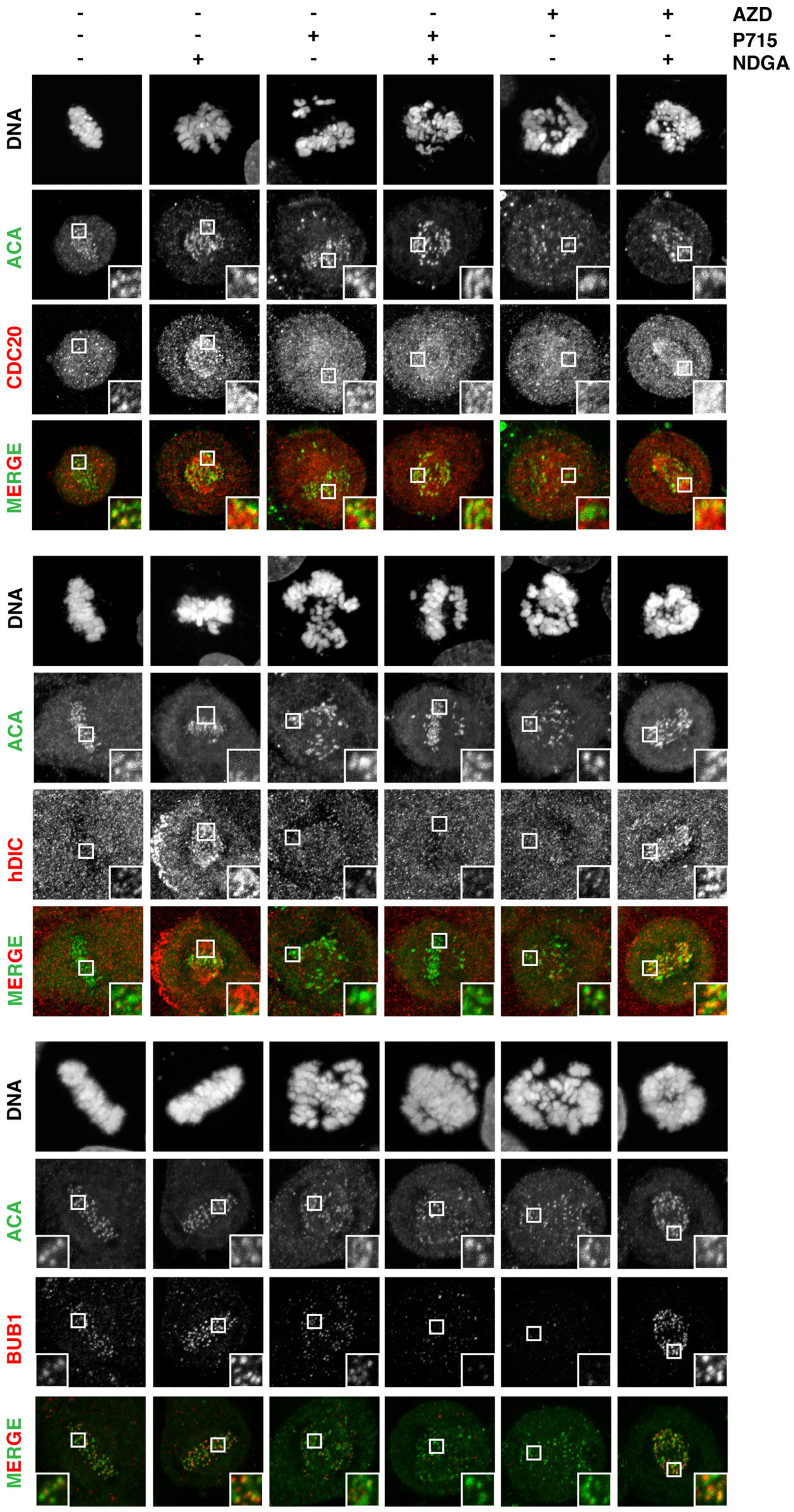

AZD
P715
NDGA
DNA
ACA
CDC20
MERGE
DNA
ACA
hDIC
MERGE
DNA
ACA
BUB1
MERGE

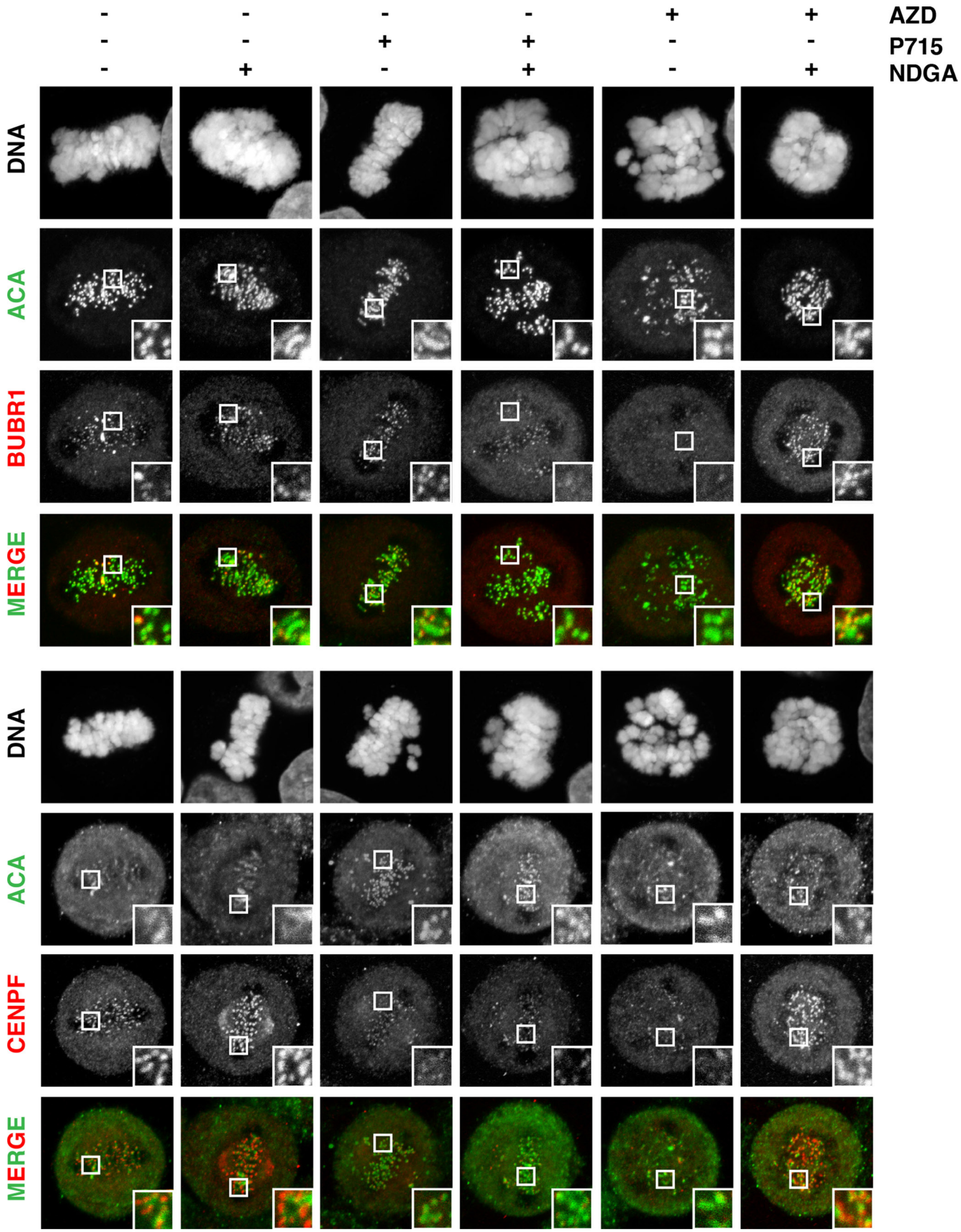

Supplementary Figure 7: GFP-MPS1 and other SAC proteins are stripped from the kinetochore following Aurora B inhibition. Immunofluorescence images of HeLa cells, showing the localisation of the SAC proteins, when treated with the indicated drugs for one hour. Note that MG132 was also added to all cells. The white boxes are enlarged to highlight kinetochores and/or centrosome staining. AZD; 0.5 μM AZD1152, P715; 1.5 μM NMS-P715.

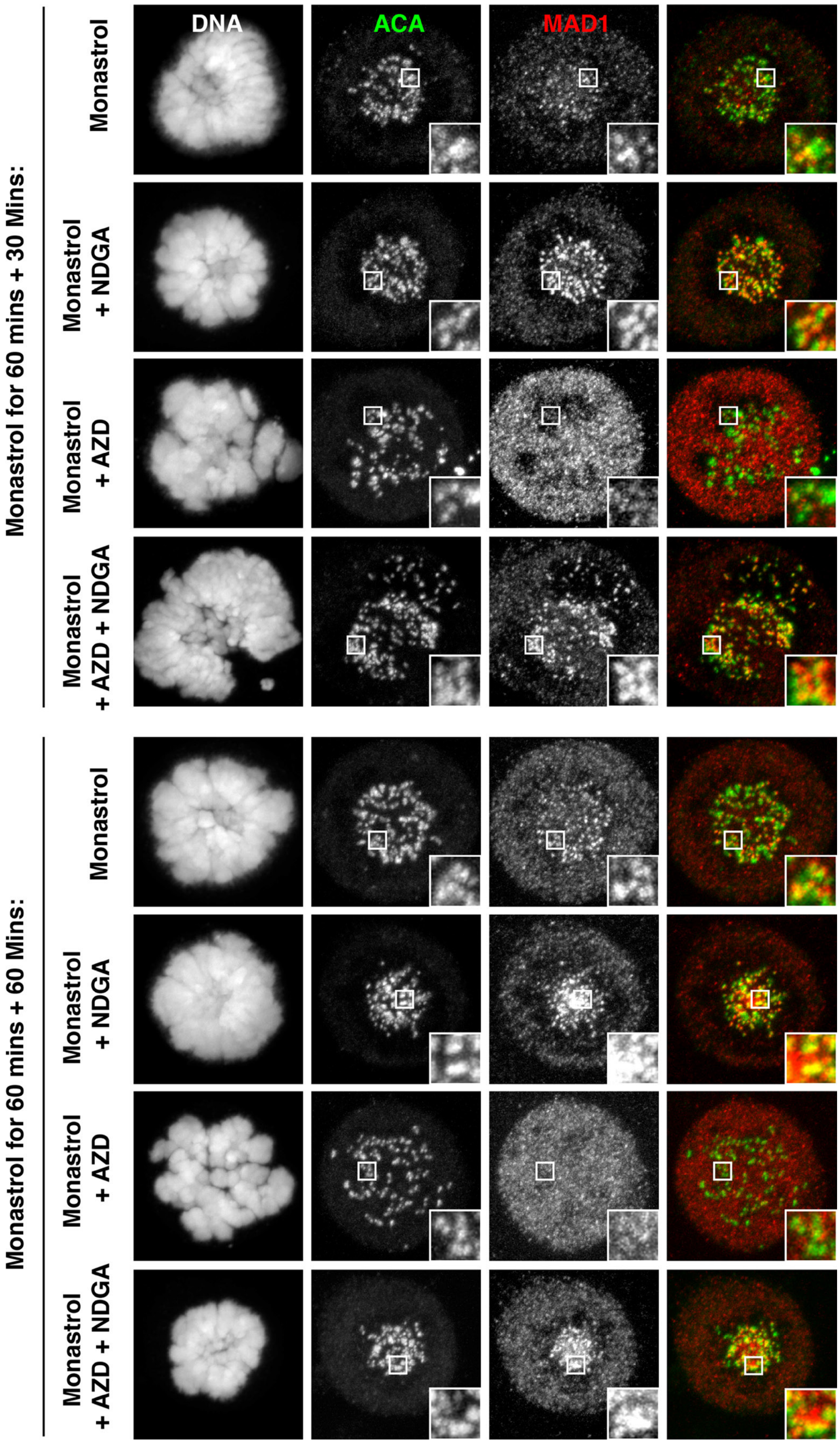

Supplementary Figure 8: Time-course of MAD1 centrosome accumulation following NDGA treatment. Immunofluorescence images of HeLa cells, showing the localisation of MAD1 when treated with the indicated drugs for the indicated time. Note that MG132 was also added to all cells. The white boxes are enlarged to highlight kinetochores and/or centrosome staining. AZD; 0.5 µM AZD1152.

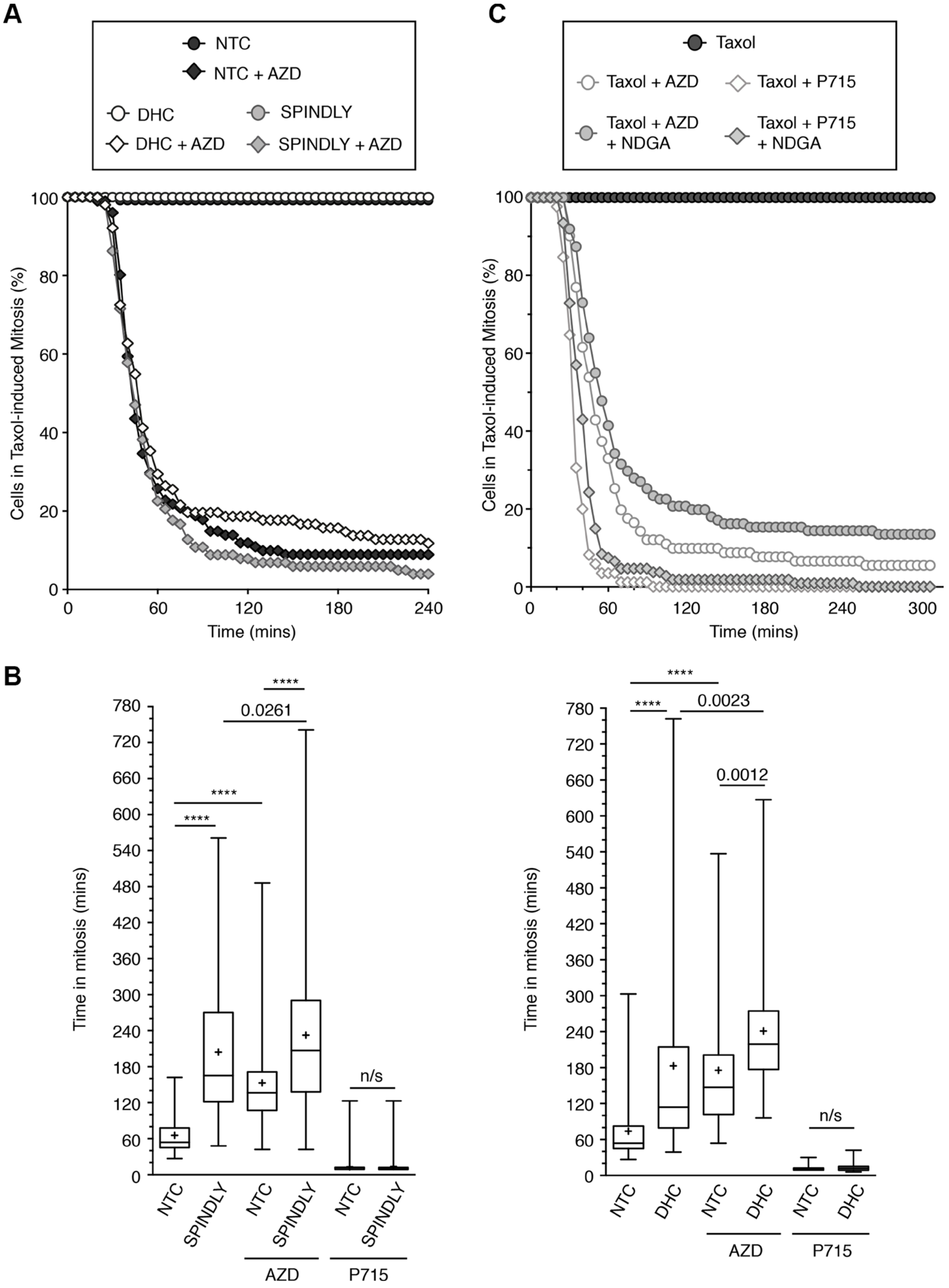

Supplementary Figure 9: Neither SPINDLY or DHC RNAi, nor NDGA can prevent SAC silencing when cells are arrested in taxol. (**A**) Line graph showing the mitotic exit of HeLa cells, following 48 hours RNAi, arrest in mitosis using taxol (tax) for 18 hours, then treated with 0.5 µM AZD1152 (AZD) at 0 mins. *N* = >100 cells per condition. RNAi (ON-TARGETplus, GE Dharmacon) was used at a final concentration of 50 nM and transfected using DharmaFECT 2 reagent (GE Dharmacon). NTC = non-targeting control, DHC = Dynein heavy chain. (**B**) Box-and-whisper plot showing the time HeLa cells (stably expressing Histone H2B-mCherry) spent in mitosis, 48 hours after non-targeting control (NTC), SPINDLY (Left) or Dynein heavy chain (DHC – right) RNAi, with addition of 0.5 µM AZD1152 and 1.5 µM NMS-P715. The boxes represent the interquartile ranges and the whisker the full range. The result was analysed by One-way ANOVA with **** indicating $p < 0.0001$ and n/s = not significant. *N* = > 65 cells per condition. (**C**) Line graph showing the mitotic exit of HeLa cells, analysed by time-lapse, arrest in mitosis in taxol (tax) for 18 hours, then treated with 0.5 µM AZD1152 (AZD), 1.5 µM NMS-P715 (P715) and 100 µM NDGA at 0 mins. *N* = > 100 cells per condition.

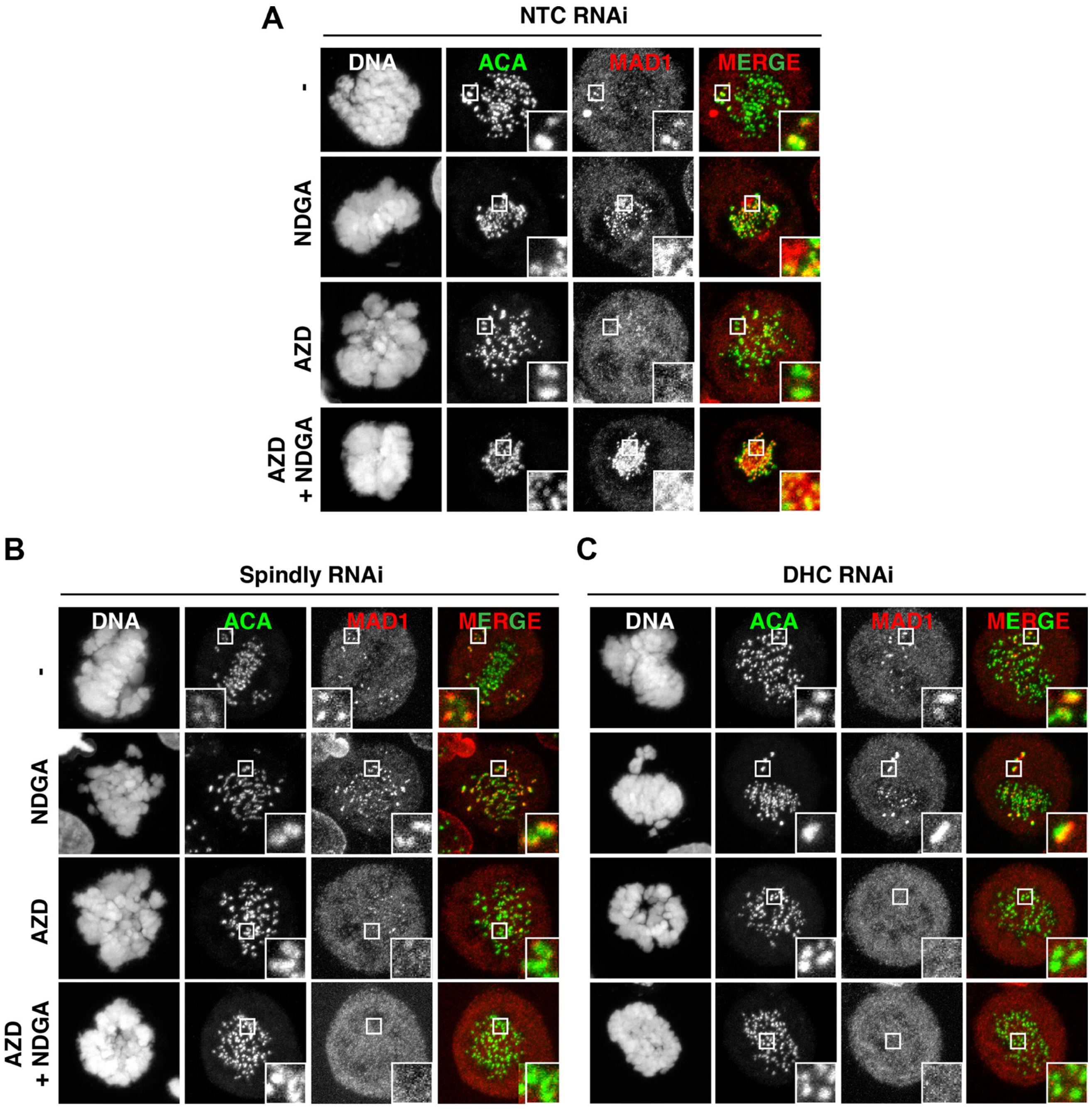

Supplementary Figure 10: SPINDLY or DHC RNAi prevents the centrosome accumulation of MAD1 following NDGA treatment. (**A–C**) Immunofluorescence images of HeLa cells, showing the localisation of MAD1, following NTC (A), SPINDLY (B) or DHC (C) RNAi for 48 hrs, and when treated with the indicated drugs, for one hour. MG132 was added in all conditions. The white boxes are enlarged to highlight kinetochores and/or centrosome staining. AZD; 0.5 µM AZD1152.

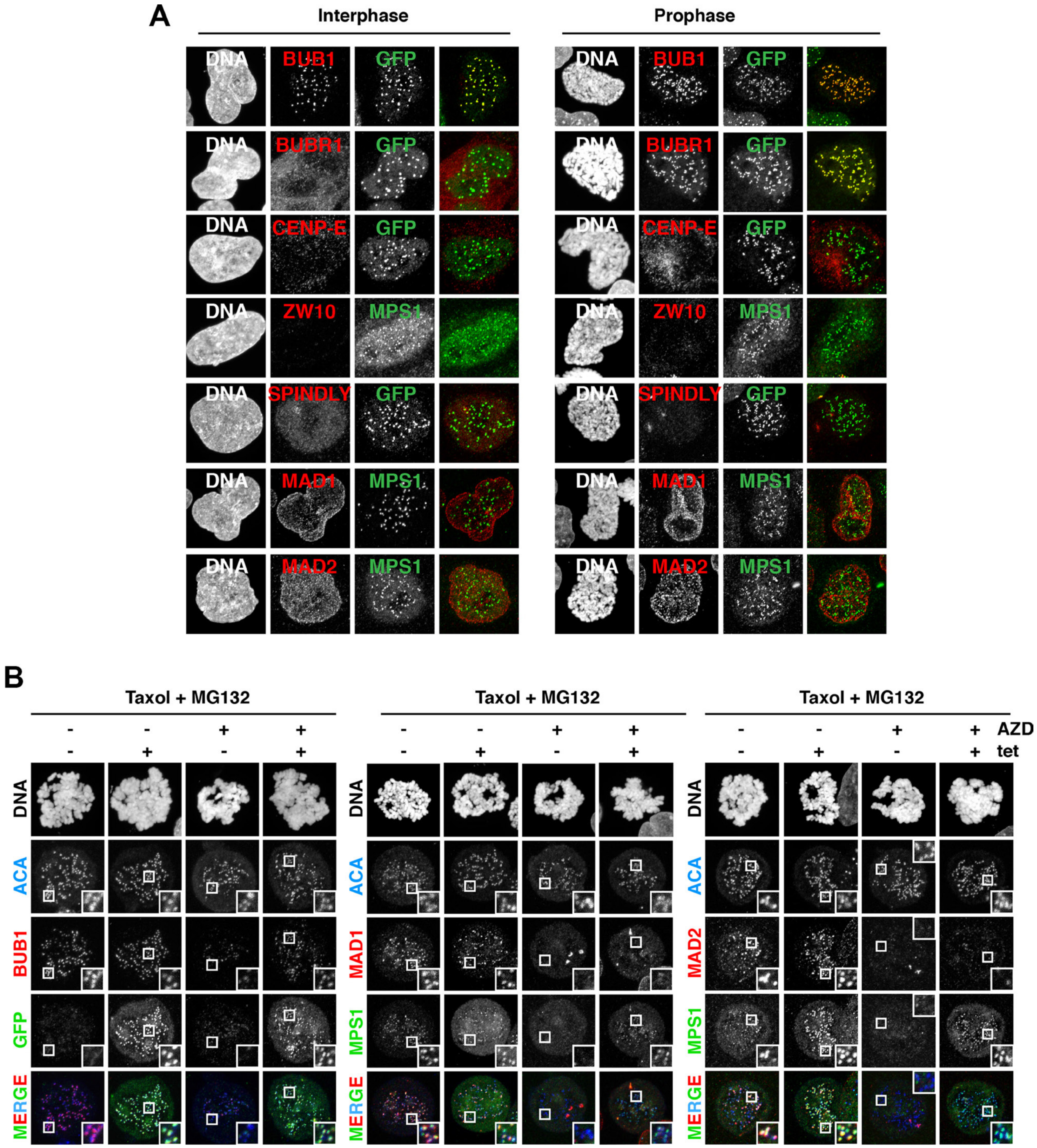

Supplementary Figure 11: GFP-MIS12-MPS1ΔN expression only causes interphase recruitment of BUB1, but cannot prevent stripping of kinetochore proteins. (**A**) Immunofluorescence images of HeLa cells to show the interphase and prophase recruitment of kinetochore proteins when expressing GFP-MIS12-MPS1ΔN. (**B**) Immunofluorescence images of HeLa cells to show the retention of proteins at the kinetochore, following expression of GFP-MIS12-MPS1ΔN and treatment with AZD1152.

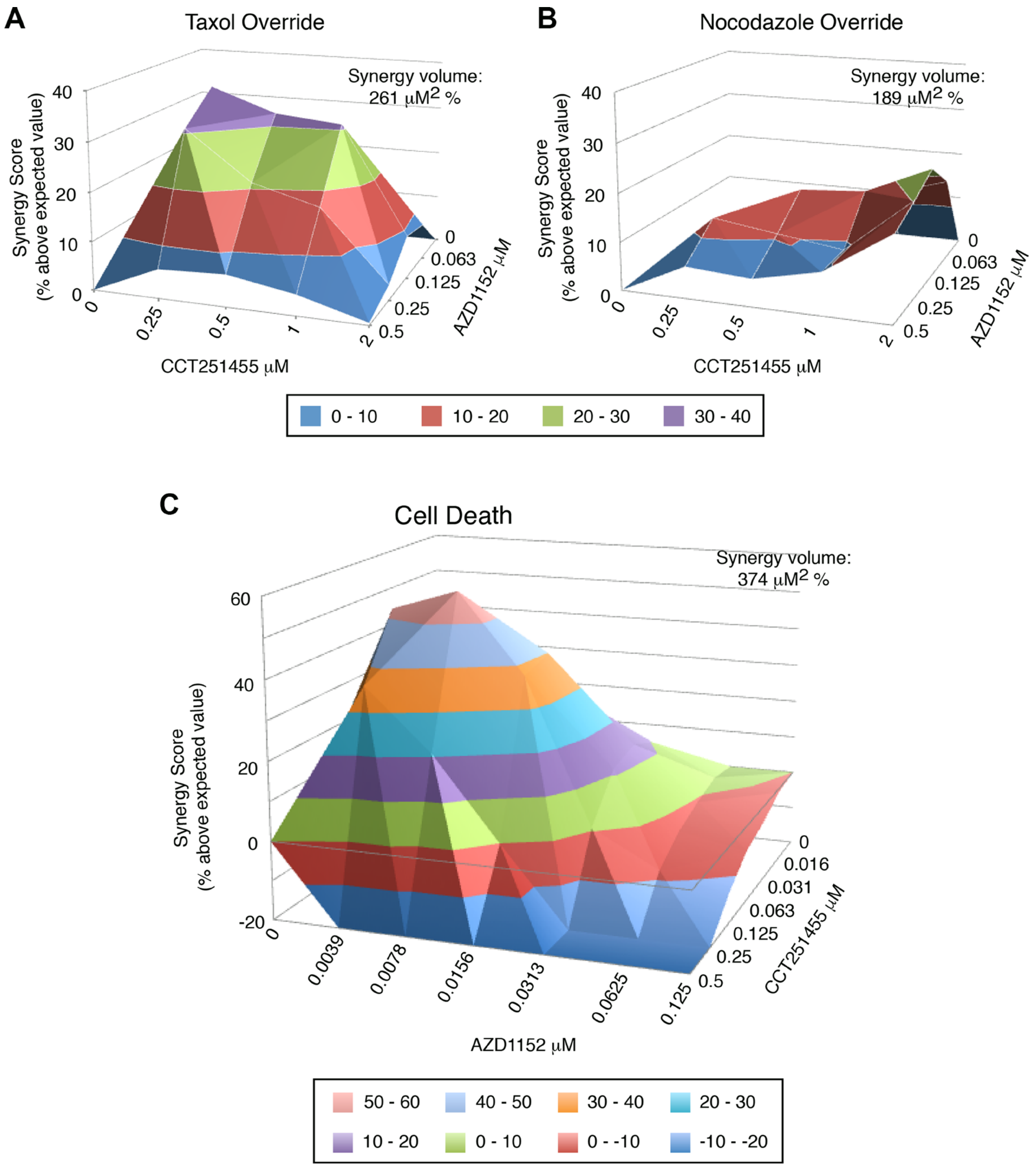

Supplementary Figure 12: CCT251455 and AZD1152 synergise to override a taxol-induced SAC and synergise in causing cell death in HeLa cells. (**A**) Synergy 3-D plot showing the synergistic override of a taxol-induced mitotic attest in HeLa cells using AZD1152 and CCT251455. The mean time spent in mitosis was used from Figure 5a and the synergy score calculated using MacSynergyII. (**B**) Synergy 3-D plot showing the override of a Nocodazole-induced mitotic attest in HeLa cells using AZD1152 and CCT251455. The mean time spent in mitosis was used from Figure 5d and the synergy score calculated using MacSynergyII. (**C**) Synergy 3-D plot showing the synergistic killing of HeLa cells when treated with AZD1152 and CCT251455. The survival fractions were used from the experiment in Figure 7a and synergy score calculated using MacSynergyII. The mean of 3 experiments is shown.

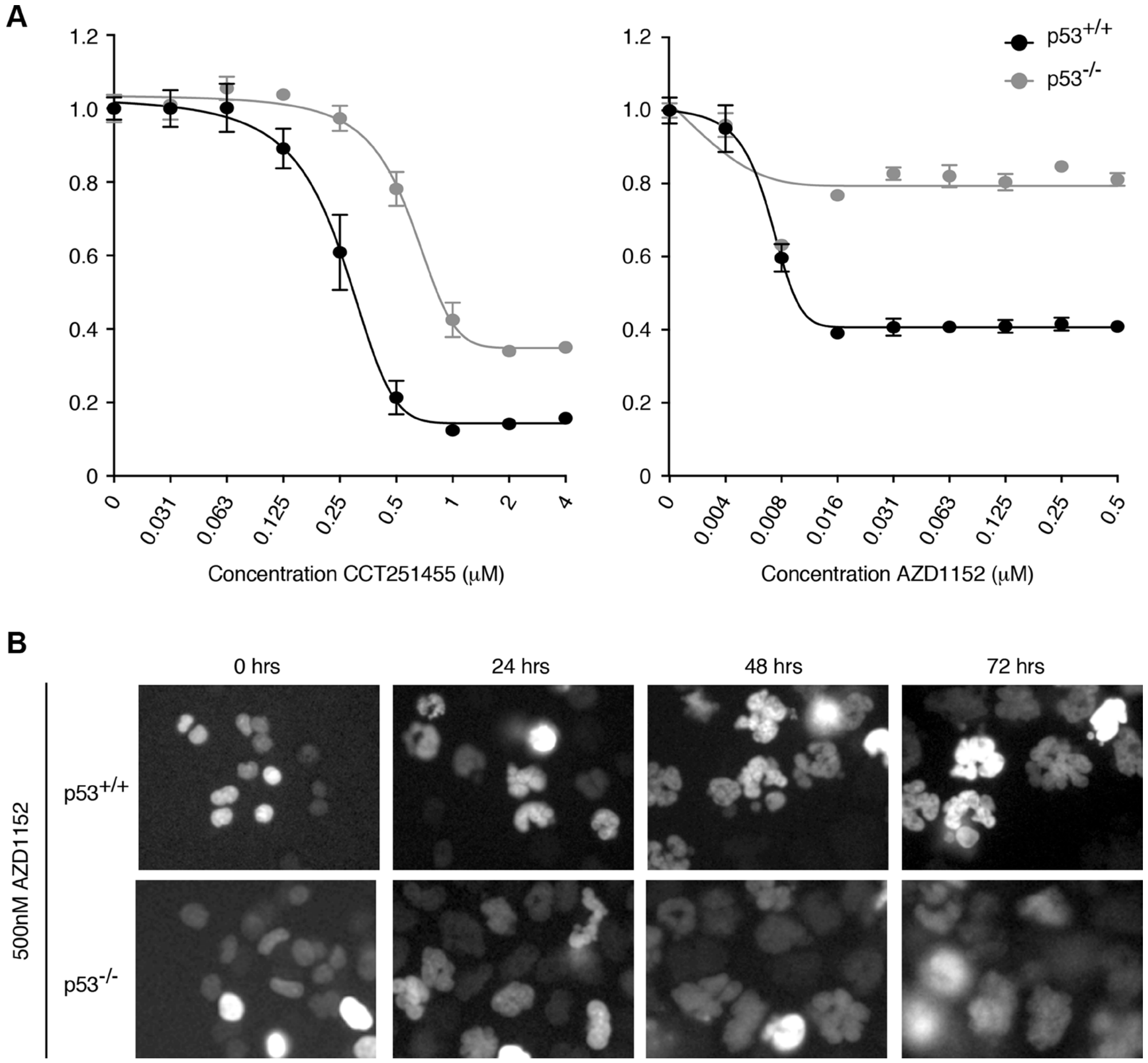

Supplementary Figure 13: The response of p53 proficient and deficient HCT116 cells to AZD1152 and CCT251455. (**A**) Line graph showing the cell viability of HCT116 cells in response to 4-day treatment with CCT251455 (left) and AZD1152 (right). The mean of three experiments +/– SD is shown. (**B**) Representative time-lapse images showing polyploidisation of both p53 proficient and deficient HCT116 cells when treated with AZD1152 over 72 hours.

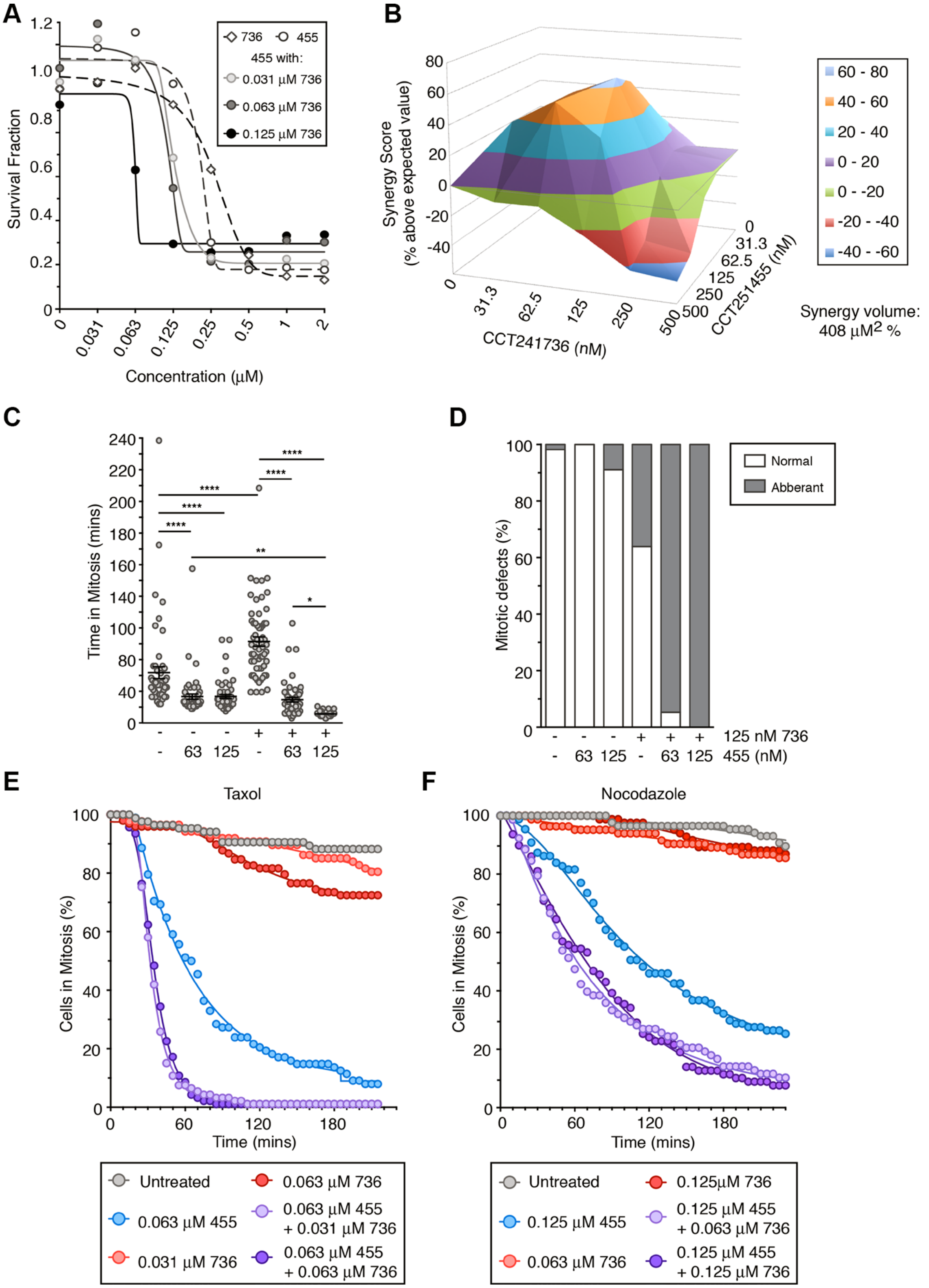

Supplementary Figure 14: CCT251455 and CCT241736 synergise in killing cells through override of the SAC. (**A**) Line graph showing the cell viability of HeLa cells in response to CCT251455 and CCT241736, alone and in combination. The mean of three experiments is shown. (**B**) Synergy 3-D plot of data from (A), showing the synergistic killing of HeLa cells when treated with CCT251455 and CCT241736. The synergy score calculated using MacSynergyII. (**C**) Scattered dot plots showing the time spent in mitosis of asynchronous HeLa cells (stably expressing Histone H2B-mCherry) in the absence and presence of CCT251455 and/or CCT241736. N = >55 cells per condition and analysed by One-way ANOVA with *** indicating $p < 0.0001$. (**D**) Bar graph quantifying the percentage of chromosome segregation errors of cells in (C). (**E–F**) Line graphs showing the exit of individual cells from a (E) taxol- and (F) nocodazole-induced mitotic arrest, following treatment with CCT251455 and/or CCT241736. Inhibitors were added at T = 0 mins and mitotic exit was visualised by time-lapse microscopy. N = >82 cells per condition.

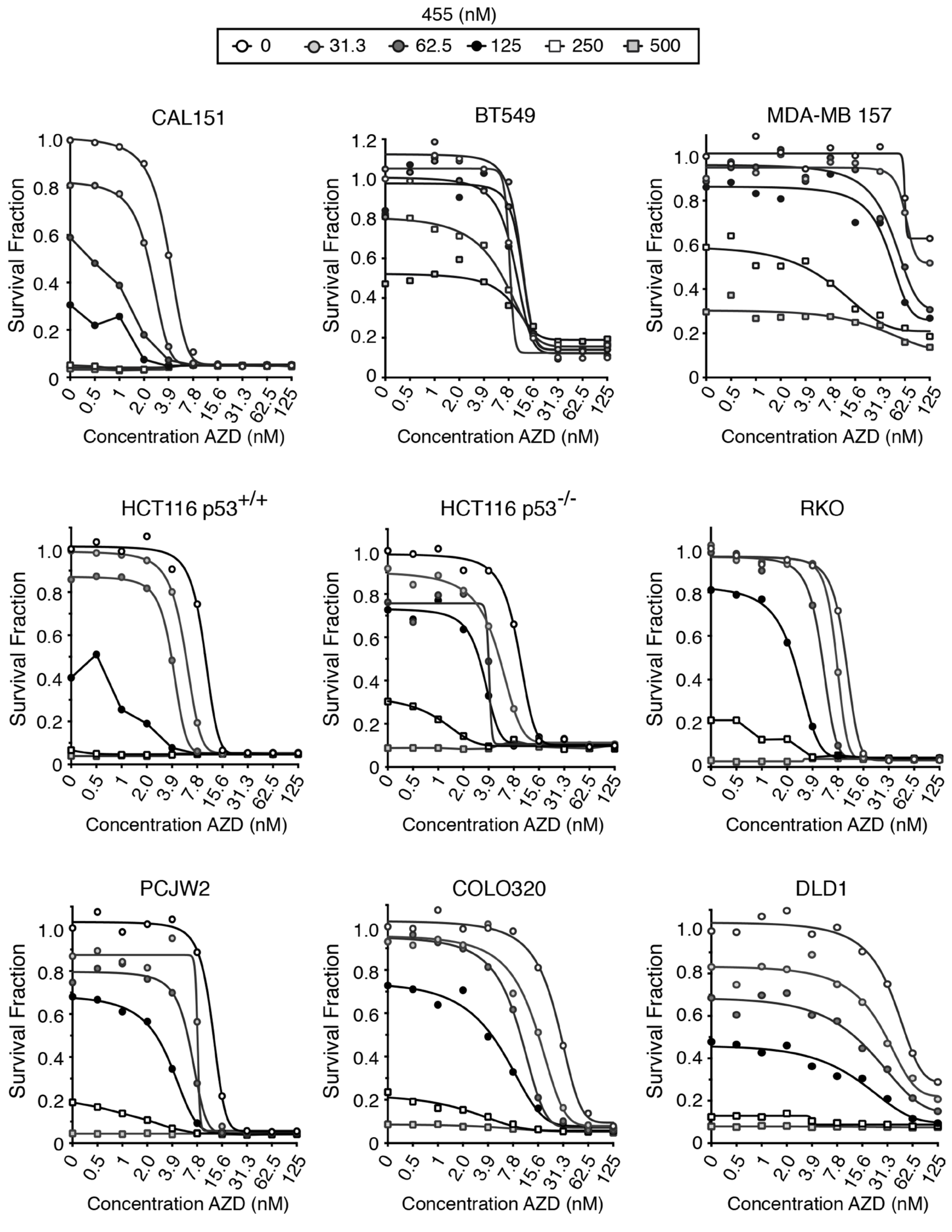

Supplementary Figure 15: CCT251455 and AZD1152 synergise in killing breast and colon cancer cell lines. (**A**) Line graph showing the cell viability of the indicated cell line in response to CCT251455 and AZD1152. The mean of three experiments is shown.
